# Identifying author heritage using surname data: An application for Russian surnames

**DOI:** 10.1002/asi.24104

**Published:** 2019-01-25

**Authors:** Maria Karaulova, Abdullah Gök, Philip Shapira

**Affiliations:** ^1^ Manchester Institute of Innovation Research, Alliance Manchester Business School, University of Manchester Manchester, M13 9PL UK; ^2^ Hunter Centre for Entrepreneurship, Strathclyde Business School University of Strathclyde 199 Cathedral Street, Glasgow, G4 0QU UK; ^3^ School of Public Policy Georgia Institute of Technology Atlanta GA, 30332‐0345 USA

## Abstract

This research article puts forward a method to identify the national heritage of authors based on the morphology of their surnames. Most studies in the field use variants of dictionary‐based surname methods to identify ethnic communities, an approach that suffers from methodological limitations. Using the public file of ORCID (Open Researcher and Contributor ID) identifiers in 2015, we developed a surname‐based identification method and applied it to infer Russian heritage from suffix‐based morphological regularities. The method was developed conceptually and tested in an undersampled control set. Identification based on surname morphology was then complemented by using first‐name data to eliminate false‐positive results. The method achieved 98% precision and 94% recall rates—superior to most other methods that use name data. The procedure can be adapted to identify the heritage of a variety of national groups with morphologically regular naming traditions. We elaborate on how the method can be employed to overcome long‐standing limitations of using name data in bibliometric datasets. This identification method can contribute to advancing research in scientific mobility and migration, patenting by certain groups, publishing and collaboration, transnational and scientific diaspora links, and the effects of diversity on the innovative performance of organizations, regions, and countries.

## Introduction

One of the uses of bibliometric research is to extricate information that is not explicitly presented in mainstream databases, such as inferring patterns of new technology emergence, the extent of novelty and originality in patents, or identifying author gender, academic age, or migration trajectories from research publications. The exposition of implicit information can increase the value of data and its explanatory power. The author name represents a data item from which further information can be deduced. For example, some analysts have used first names to assign gender (Meng & Shapira, 2010; Thelwall & Kousha, 2014). Surname data have been used to infer characteristics such as ethnic origin (Webster, 2004), but such data have found relatively little use in bibliometric scholarship. This article seeks to extend the use of surname data to identify the national heritage of researchers in bibliometric datasets by offering an approach that uses the morphological regularities of names.

Despite ever‐increasing global mobility, the distribution of surnames still has clear geographic patterns both between and within countries (Colantonio, Lasker, Kaplan, & Fuster, 2003). For many categories of names, it is possible to ascertain the probable heritage of their holders. Existing name‐based identification methodologies predominantly use dictionaries to probabilistically assign names to ethnic, language, or country groups. Dictionary‐based methods are appropriate for name types with low variability, such as Chinese names, and with names associated with “broad” ethnic groups such as “Hispanic” (in US classifications). For naming traditions with higher variability, these methodologies are inhibited by low sensitivity and low precision. In bibliometric datasets specifically, testing the effectiveness of these methods has been limited because of the lack of reliable data for results comparison.

The objective of this article is to develop a methodology based on the approach of identifying names through morphological regularities. The logic of this method can be used to identify a variety of surname types. In this article, we applied the general principle on Russian surnames, with the aim to identify Russian heritage. Although Russian surnames share some traits with Eastern European family names, the magnitude and impact of Russian research is much higher than that of other Slavic nations, which makes it a suitable case for a bibliometric dataset. Following an established methodology for name research, we developed an identification procedure conceptually and then used the 2015 ORCID (Open Researcher and Contributor ID) public data file to construct a control dataset using a randomized proportionate undersampling method, in which the effectiveness of the procedure was tested.

This article makes three contributions. First, the method described here can be adapted for any group of surnames, provided that group has identifiable morphological regularities. Second, the method aims to identify heritage, and not ethnicity. In fact, as we specify later, most studies that address the issue of ethnicity, which is a biological feature, actually mean heritage, which is a sociocultural trait. Third, we outline areas of interest in science and innovation that would benefit from using this method: (a) research on scientific mobility, (b) the role of persons with a particular heritage in global networks and interactions, (c) research on the impact of heritage diversity on creativity and innovation, and (d) the influence of heritage on academic behavior and strategies.

The article is structured as follows. The next section provides a background review on the use of surname data to infer the demographic properties of their holders. We then outline the method and data. Subsequent sections review the testing of the identification procedure and demonstrate the usefulness of the procedure by applying it to the dataset of ORCID users in 2015. The concluding section discusses the usefulness of the procedure, notes the limitations, and outlines its further applications in bibliometric research.

## Background

The longstanding tradition of using name data to infer the implicit characteristics of their holders spans multiple disciplines in both the social and the natural sciences. In physical anthropology, human population biology, and genetics, the occurrence of the same surname in couples has been studied to analyze the persistence of naming patterns in geographical regions to infer inbreeding and genetic similarities. These studies have found that surname clusters are characterized by low genetic distances (Rodriguez‐Larralde, Gonzales‐Martin, Scapoli, & Barrai, 2003) and that patterns of social and health outcomes linked to genetic structures can be traced via surnames (Kandt, Cheshire, & Longley, 2016).

In the social sciences, the scope of research that uses surname data is also broad. Research in history, sociology, and linguistics has explained the historical and social context of names, such as the socioeconomic determinants of naming (Bloothooft & Onland, 2011). Another body of knowledge aims to explain how the properties of names change the behavior of individuals or groups who encounter holders of those names. This research explains phenomena such as nominative determinism—the likelihood of a person to pursue and be perceived as successful at a particular career if their name resembles that career (Gueguen & Pascual, 2011), and nominative discrimination—the likelihood of not receiving certain privileges because of a name (Hogan & Berry, 2011). Bibliometric data have been used in this vein to study alphabetical favoritism—the increased likelihood that scientists with earlier alphabetical initials will receive higher reputational rewards (Einav & Yariv, 2006).

Surnames can be used to infer the characteristics of certain name holders that are otherwise unobtainable in datasets. Although variations exist among different naming traditions, it is possible to associate traits such as a person's social class or faith from his or her name (Clark & Cummins, 2015; Susewind, 2015). Name data can be reliably used to estimate ancestry in multiethnic populations so as to assess issues such as access to healthcare, long‐term population change, and ethnoregional disparities (Mateos, 2007).

Most of these latter studies infer ethnicity by developing name dictionaries. Their efficiency is estimated by comparing probabilistic assignment with alternative reliable ethnicity data in testing scenarios. The efficiency of dictionaries varies significantly between ethnicities. “Hispanic” names obtain more than 85% recall (Wei, Virnig, John, & Morgan, 2006), Chinese and Korean name lists return recall and precision on average above 70% (Kim, Lauderdale, Shin, & Lee, 2014; Quan et al., 2006), whereas such efficiency measures for “Asian American” or “Arab” ancestry lists do not exceed 60% (El‐Sayed, Lauderdale, & Galea, 2010; Lauderdale & Kestenbaum, 2000).

Among recent methodological developments, combined geo‐coding and surname methodologies report the best results for multigroup classifications (Elliott et al., 2009). However, the vast majority of surname research in health and population studies use US data and broad US‐defined ethnicity categorizations (which include “Hispanic” or “Asian”).

In bibliometric scholarship, the use of name data to extrapolate demographic characteristics is limited. Bibliometric research that uses names also relies mainly on dictionaries. Some studies have sought to distinguish a particular type of surname by using small datasets delineated by country, discipline, or population subgroup. Here, name data serves mainly as an indication of ethnicity. The scope of issues explored spans the productivity of immigrant inventors (Kerr & Lincoln, 2010), global technology transfer (Kerr, 2008), ethnic coauthorship (Freeman & Huang, 2015), mobility patterns within or across countries (Lewison & Kundra, 2008), and the identification of minority groups in scientific populations and their contributions (Kissin & Bradley, 2013). Kerr's name‐matching program (Kerr, 2007) is the only method that has attempted to assign names to multiple ethnic categories. It combines probabilistic assignment and manual coding within the US patent data, but only reports recall scores and does not address surname assignment overlaps.

The adaptation of named entity recognition methods from information science to use in bibliometrics has also been limited. Despite promising attempts, empirical applications in bibliometrics have not been as effective as those in other fields (Robinson‐Garcia, Noyons, & Costas, 2015). With respect to Russian‐named entities, significant progress has been reported (Mozharova & Loukachevitch, 2016; Starostin et al., 2016). However, these methods have almost exclusively been applied to texts in Cyrillic, aim to extract named entities from unstructured text, and do not distinguish between different types of named entities within a certain class (for instance, surnames with different heritage).

Most bibliometric datasets are naturally limited in that they do not have the capacity to test the power of surname‐based inference. Published works that report information retrieval scores prioritize recall over precision, and that affects the applicability of the method to large datasets. Dictionary‐based matching identifies only the most “popular” names and does not account for name and spelling variability.

One alternative to using dictionaries is to use the structure of names to infer the demographic properties of their holders. For example, Lewison (2001) identified female researchers from Iceland by selecting surnames ending in “*dottir.”* To date, the identification of population groups in bibliometric datasets based on surname structure has found limited application, despite its potential benefits.

## Research Design

### *Objective*

Dictionaries, by themselves, cannot be reliably used to infer the heritage of populations with high name variability. We develop a method that instead takes advantage of morphological regularities of certain surname types, thus complementing dictionary‐based identification methods.

As a test group, Russian surname morphology is used. Russian surnames have extremely high variability. Dictionary‐based methods only identify the most common names. Russian surnames have morphological regularities, which makes them a good empirical set to test the strength of this identification method.

### *Terms Operationalization*

Most studies in bibliometrics analyze the behavior of groups based on their shared *heritage*, not on ethnicity, despite frequently using the language of “ethnicity.” Surname‐based identification methods originated within genetics, evolutionary biology, and general population research. Scholars from these disciplines use surname data to identify the ethnic origin of populations in order to assess the heightened health risks associated with particular ethnicities. Although bibliometric studies borrow some of these methodologies for ethnicity identification, they usually use ethnicity as a proxy for particular traits of behavior, values, and social norms among researchers of a given origin. Shared heritage is a preferable classification because heritage more plausibly influences an individual's scientific behavior, not ethnicity, which is a biological feature. Heritage links memory, language, and place with the construction of identity and communities (Smith, 2006). It refers to shared values, attitudes, skills, and tacit knowledge, whereas ethnicity only implies genetic similarity.

In this research, we extend the notion of heritage by associating it with the concept of human capital. Knowledge, skills, and other forms of noneconomic capital accumulated by each person become a part of that person's heritage, in that they have an impact on the person's identity, which, in turn, can influence their behavior. Studying heritage refers more to accounting for those intrinsic and specific features of cultural capital within social groups that make the groups distinctive in how they approach problems and look for solutions.

It follows that in science and innovation, each specific type of heritage can be associated with specific configurations of scientific and technical (S&T) human and social capital (Bozeman, Dietz, & Gaughan, 2001). Heritage indicates a particular identity, behavior, belonging to communities, embeddedness in certain networks, and other social characteristics that influence a person's behavior. For example, when Borjas and Doran (2012) studied the influence of the post‐1992 influx of Soviet mathematicians on the organization of US mathematics, they examined productivity patterns based on topic selection, collaboration strategies, and the publication practices of the two groups. All of them stemmed from the differences between the US and Soviet heritage in mathematics. Similarly, when Freeman and Huang (2015) used surname data to identify “Chinese ethnicity,” they aimed to study copublication strategies based on shared topic interests, shared tacit knowledge, and shared networks, all of which are manifestations of shared “Chinese” academic heritage.

When ethnicity and heritage do not coincide, it becomes important to distinguish them. For example, *Soviet heritage* can be conceptualized as a shared legacy of a command‐and‐control research system dominated by the Academies of Sciences that had little interface with industry and featured divisions between research and teaching (Vucinich, 1984). These specific traits of how research was organized influenced the type of human and social capital, skills, attitudes, and values of scientists and engineers who were trained in the USSR. The impact of Soviet heritage is still felt across the countries of the former Soviet Union and the Eastern Bloc (Radosevic, 2003), but it has nothing to do with ethnicity.

Similarly, the contemporary Russian research system is heavily path‐dependent on its Soviet predecessor (Karaulova, Gök, Shackleton, & Shapira, 2016; Karaulova, Shackleton, Liu, Gök, & Shapira, 2017). Russia is a multiethnic and multifaith country, yet in research and innovation it is appropriate to discuss post‐Soviet *Russian heritage*. Specific normative attributes, skills, and strategies related to productivity, collaboration behaviour, career development patterns, technology transfer and patenting distinguish Russian scientists.

Heritage is not explicitly codified in publication data records and, therefore, has to be inferred. This research uses surname data to infer heritage and compares the findings with ORCID profile data on education and affiliation history in Russia. We test the proposition that Russian heritage can be inferred from the surname and first name.

We assume that ORCID users with an education and employment history in Russia are primarily likely to be Russian nationals with Russian names and heritage. Multiple observations support this assumption. First, before the breakup of the Soviet Union, holders of Russian surnames were mostly clustered in the territory of Russia (Revazov, Paradeeva, & Rusakova, 1986). In research specifically, the Russian science system has remained relatively nationally bounded. Russian is the dominant language of academic publications, and academic job mobility is very limited. Although comprehensive statistical data about non‐Russian nationals employed in Russian research is not available, evidence indicates that foreign‐born student and researcher numbers remain low, despite policy efforts (Sivak & Yudkevich, 2008). Therefore, it is reasonable to assume that Russian science employs mainly Russian scientists. Based on this assumption, we validate the efficiency of surname‐based identification by comparing its results with the affiliation country of ORCID users.

This research uses *name data* to infer heritage. Both given name and surname data are used in the procedure. Surname data for the procedure does not need to be written in any particular format, as long as it is searchable separately from non‐name data in each record. Surname data are complemented by given name data, where available. Russian given names are diverse: they encompass Orthodox Christian names of biblical, Greek and Latin origin, Slavic names, and names specific to ethnic and religious minority groups. After the 1918 Revolution, practices of name import and name creation emerged (Petrovsky, 1966). Given (or first) name data by itself is unsuitable for identifying Russian heritage. However, Russian variants of many Christian and Slavic given names have a specific spelling, and we use them as an extra tool to eliminate false‐positive results, thus complementing the surname‐based method.

### *Data*

The data for this study came from the Open Researcher and Contributor ID (ORCID) database, which is an open international initiative that aims to build a registry of unique researcher identifiers on a global scale (see: https://orcid.org/). The ORCID identifier is a nonproprietary alphanumeric code. It provides a unique and persistent identifier for each individual user, which they can then assign to their funding bids and their publication metadata. In 2017, ORCID included over 3.1 million individual members (Haak, 2017).

ORCID is increasingly recognized as a cross‐platform identifier and is integrated with mainstream repository and reference systems, such as PLoS, the Royal Society, SCOPUS, ProQuest, and the Web of Science.^1^ Early exploration of ORCID's value in bibliometric research suggests that, although with some limitations, ORCID data can provide a way to solve long‐standing issues such as authorship ambiguity (Youtie, Carley, Porter, & Shapira, 2017).

A data file, which aggregates all public data in the ORCID registry associated with each ORCID member, is made available annually. In this work, the 2015 ORCID public data file was used as a dataset for testing the surname‐based heritage inference approach. After cleaning, the dataset contained 307,459 public ORCID identifiers, which were associated with 294,746 author names. For each identifier, data on the following affiliation types and history were available: employment and education; position; dates of employment and education; the name of the institution; and its location (city, region, and country).

**Figure 1:**
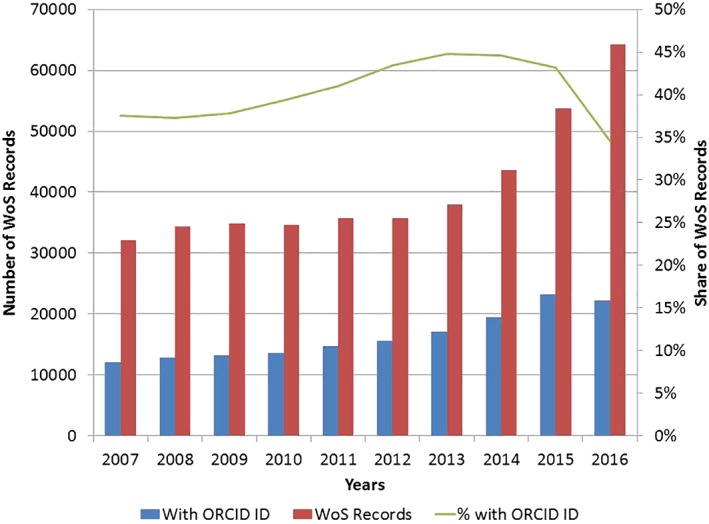
Publication records with ORCID ID identifiers listing affiliations in Russia (source: Web of Science, *N* = 406,830). [Color figure can be viewed at http://wileyonlinelibrary.com]

At the time of the study, Russia ranked 12^th^ among countries by ORCID user population: 8,799 users (2.98% of all ORCID users) reported that they either worked or studied in Russia. In terms of coverage, the share of scientific publications with Russian affiliation addresses that contain ORCID identifiers in the Web of Science has been increasing and peaked at ~45% in 2014 (see Figure 1).

## Method

The total number of Russian surnames exceeds 15,000 (Balanovskaya, Solovyova, & Balanovsky, 2005). The semantic origins of Russian surnames are highly variable, which precludes composing an exhaustive list. Similar to other Slavic naming traditions, a Russian surname can be composed of almost any word by adding one of several specific suffixes. As a result, surnames in Russia are continuously being created: for example, both “Lenin” and “Stalin” are made‐up surnames, which were initially used as aliases.

A suffix is a linguistic unit that is placed at the end of the word. The majority of surname suffixes indicate belonging to a family (patronym), place, or profession. In the Russian language, female surnames, with minor exceptions, are characterized by adding the ending *–a* after the suffix. The method proposed in this article takes advantage of these morphological regularities to identify Russian surnames.

Boris Unbegaun's (1972) landmark work, the most comprehensive Russian surname etymology study to date, provides a morphological classification of Russian surnames. The “overwhelming majority” (Unbegaun, 1972, p. 2) of them have one of the three most popular patronymic suffixes *–ov, ‐ev,* and *‐in*. Minority types of Russian surnames include surnames with other patronymic suffixes, adjectival surnames (derived from adjectives), substantival surnames (derived from nouns), and surnames of various foreign origins (see Table 1). All patronymic and adjectival suffixes have strong regularities, which makes them easily identifiable. Identifying them comprises the “base rule” of the procedure.

**Table 1:** Types of Russian surnames (adapted from Unbegaun, 1972).

Surname type	Definition	Examples	General popularity
Patronymic/metronymic	Surnames are derived from a name, place, or a profession. Low variability.	Ivanov Nikitina Vyazemskiy	Overwhelming majority
Adjectival	As above, derived from adjectives. Low variability.	Chernykh	Rare
Substantival	Surnames derived from nouns. High variability.	Medved Golub	Negligible
Surnames of foreign origin	Russianized surnames of foreign origin. Varying popularity and some suffix variability depending on the origin.	Landau Bidon'ko	Rare

Substantival surnames have variable morphologies, which do not adhere to any particular rule. Surnames of foreign origin are regular but also variable—some have regularities in morphology, and some do not—but it is difficult to estimate their pervasiveness in the Russian population.

Three additional considerations were addressed during the method development: database bias, territorial bias, and bias related to the lack of updated information on Russian surnames.

First, we considered the relative frequency of different surname types (as presented in Table 1). With respect to people in scientific professions, the presence of certain types of surnames may vary from that of the general population. Irregular surnames, especially among leading scientists, may be more frequent. For example, among the 12 Russian/Soviet Physics Nobel Prize Laureates in the 20^th^ century, seven had “non‐Russian” surnames. The roots of this overrepresentation may go as far back as the foundation of Russian academic science in the 18^th^ century and the persistence of Germanic scientific dynasties (Lipski, 1953). This bias is addressed in the procedure by inspecting the names of the top 1% most‐cited scientists in the Russian Science Citation Index (RSCI).^2^ In February 2017, RSCI contained the names of 813,069 scientists. The names of 8,130 top‐cited scientists were checked for nonconventional surnames.

Second, there is geographic diffusion and overlapping in Russian and other Eastern European naming traditions. Some popular Russian names have naming patterns stemming from countries that are not currently within Russia's borders. Conversely, Russian surname morphology has been adopted in Central Asian countries, which now exist as separate nations. Some Eastern European countries, such as Poland and Bulgaria, have patronymic suffix‐based naming morphology that is similar to the Russian naming morphology. This territorial bias is addressed methodologically by including a selected sample of Russian surnames with the origin in Soviet countries in the identification procedure and by utilizing first name data to distinguish, where possible, between Russian and non‐Russian given name and surname combinations.

Third, the key name classification source (Unbegaun, 1972) for this research is dated, and more recent studies are inadequate for providing reliable data on Russian surname morphology. Therefore, the identification procedure was developed and tested in a sequential way to ensure the validity of each component.

**Figure 2:**
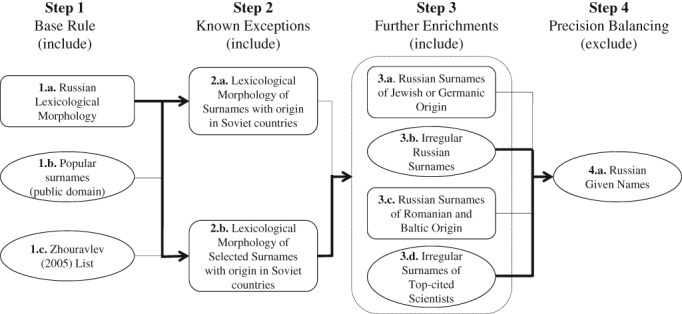
Russian heritage identification procedure sequence. Note: Rectangle shapes indicate a rule‐based search query; oval shapes indicate a dictionary‐based search. A dotted rectangle signifies mutually nonexclusive selection; bold lines signify choices made based on an *F*‐measure increment when applied to the testing dataset (source: authors).

The resulting Russian heritage identification procedure uses surname data and, when possible, first name data, and consists of four steps (Figure 2).^3^ The validity of each step is sequentially tested. Sequential construction and testing of the procedure allows precision and recall tracking of its constituent elements and of the alternatives. All steps in the procedure components use regex to identify relevant terms.

In implementing the procedure, we first identified authors whose names satisfied the “base rule.” For this opening step, we considered and tested three different alternative sources. (1.a.) Russian lexicological morphology, a rule‐based identification of Russian patronymic and adjectival surname suffixes, was tested against more conventional dictionary‐based methods: (1.b) a list of popular Russian surnames collected from the public domain, and (1.c.) a list of popular Russian surnames composed by Zhuravlev (2005).

In the second step, we identified “known exceptions” regarding the geographical bias related to the diffusion of names from the countries of the former Soviet Union, which might be origins for scientists currently working in Russia. Regarding popularity, Soviet names constitute the largest minority of “foreign” surnames in Russia but are also frequent in the countries they originated from, which are now separate nations. Using morphological regularities of “Soviet” surnames, we tested two alternative options: (2.a.) a near‐exhaustive suffix list and (2.b.) a more limited list of the suffixes popular in Russia specifically.

The third step explored possible “further enrichments” to the procedure concerning the database bias and with respect to various name minorities. Unlike Steps 1 and 2, which tested the efficiency of the alternatives against each other, Step 3 examined incremental improvements of the identification procedure, and all options that contributed to the improvements were used in its final version. This step aimed to identify: (3.a.) Russian surnames of Jewish or Germanic origin with a rule‐based query; (3.b.) irregular Russian surnames from a dictionary based on Unbegaun (1972) and information from the public domain; (3.c.) specific Russian surnames of Romanian and Baltic origin with a rule‐based query; and (3.d.) top‐performing scientists with irregular surnames, irrespective of origin, from a dictionary composed using the RSCI data.

After the selection of Russian surnames in Steps 1 through 3, we disambiguated first names by using (4.a.) a list of Russian given names retrieved from the public domain. All first names that were not identified as Russian (or when first name data was missing) were excluded from the identification results.

### *Control Dataset and Testing Criteria*

The ORCID dataset is imbalanced in that only about 3% of ORCID users report an address of work or study in Russia. Therefore, the procedure aimed to identify the very small share of records in the data as “ORCID users with Russian heritage.” Imbalanced datasets present methodological difficulties (Chawla, 2005) because attempts to increase recall of the minority class disproportionately affect the number of retrieved false positives.

With this limitation, to assess the effectiveness of the surname‐based identification procedure, we constructed a control dataset using an undersampling method. Undersampling reduces the need to control for the imbalanced impact of precision and recall on the overall measure (Drummond & Holte, 2003). Although undersampling methodologies ignore majority class qualities (Liu, Wu, & Zhou, 2009), we address this by sampling majority class surnames in proportion to their representation in the whole dataset.

Overall, 1,000 Russian surnames and 1,000 non‐Russian surnames were sampled. The former is a random selection of authors with an affiliation history in Russia. The names were manually inspected to ensure their true‐positive nature. Non‐Russian surnames were randomly selected from pools of names with a registered affiliation in a country other than Russia according to that country's popularity in the dataset (see Table A1 in the Appendix). False‐negative results for the majority class were excluded from the test dataset.

The effectiveness of data retrieval in the procedure was tested as an increment of the *F*‐measure through the sequence of steps probing alternative options. *F*‐measure is a weighted harmonic mean score that reflects the trade‐off between recall and precision of information retrieval (Buckland & Gey, 1994). The *F*‐measure is routinely used to assess the effectiveness of information retrieval in large datasets. The binary principle of relevant results selection in this procedure also makes the *F*‐score the best accuracy measurement tool.

For each step and each alternative of the classification procedure, the *F*‐measure, precision, and recall were calculated as defined in Equations 1, 2, and 3. TP represents true‐positive results, FP represents false‐positive results, and FN represents false‐negative results. The decisions about the heritage identification path were made based on *F*‐measure increments.

(1) $$\text{Precision}\;\left(P\right)=\frac{\mathrm{TP}}{\mathrm{TP}+\mathrm{FP}}$$

(2) $$\text{Recall}\;\left(R\right)=\frac{\mathrm{TP}}{\mathrm{TP}+\mathrm{FN}}$$

(3) $$F-\text{measure}\;\left(\mathrm{FM}\right)=2\times \frac{P\times R}{P+R}$$

The next section presents the results of the surname‐based classification procedure testing in the control dataset. The best identification sequence was then applied to the full ORCID dataset.

## Results

### *Procedure Testing Results*

We tested the precision and recall of alternative variants of surname identification in a sequence of four steps (refer to Figure 2). The results are summarized in Table 2.

**Table 2:** Test dataset results (source: ORCID, calculations by the authors; *N* = 2000).

Rule	Precision	Recall	*F*‐measure	Change in *F*‐measure	Decision
**Step 1: Base Rule**					
1.a. Russian Lexicological Morphology	91.05%	84.4%	87.60%		Use
1.b. Popular surnames (public domain)	97.45%	30.6%	46.58%		
1.c. Zhuravlev (2005) List	100%	27.3%	42.89%		
**Step 2: Known Exceptions**					
2.a. Lexicological Morphology of Surnames with Origin in Soviet Countries	80.87%	93.4%	86.68%	1.08% increase from 1.a.	
2.b. Lexicological Morphology of Selected Surnames with Origin in Soviet Countries	90.36%	90.9%	90.63%	3.03% increase from 1.a.	Use
**Step 3: Further Enrichments**					
3.a. Russian Surnames of Jewish or Germanic Origin	76.54%	93.3%	84.9%	5.73% decrease from 2.b.	
3.b. Irregular Russian Surnames	90.76%	95.3%	92.98%	2.35% increase from 2.b.	Use
3.c. Russian Surnames of Romanian and Baltic Origin	90.27%	90.9%	90.58%	0.05% decrease from 2.b.	
3.d. Irregular Surnames of Top‐Cited Scientists	89.45%	92.4%	90.90%	0.27% increase from 2.b.	Use
3.e. Combined Identification with the two selected “Further Enrichments”	89.87%	96.7%	93.16%	2.53% increase from 2.b.	Use
**Step 4: Precision Balancing**					
4.a. Russian Given Names	98.12%	94%	96.02%	2.86% increase from 3.e.	Use

In the first step, identification of Russian heritage names based on lexicological morphology (1.a) was the most effective alternative. FM for the rule‐based identification was 87.6%, compared with FMs below 50% for the dictionary‐based methods (1.b. and 1.c.). Name dictionaries had very high precision, but were at a disadvantage regarding recall. Therefore, the rule‐based lexicological morphology identification was used in the procedure for the subsequent testing.

Overall, an *F*‐measure of 87.6% attained in the first step is comparable to the best identification results reported by past research. However, because the ORCID dataset is highly imbalanced and we aimed to identify a minority class of ~3%, we determined that higher *F*‐score was required.

In Step 2, both options for identifying Russian surnames of Soviet origin affected recall positively and affected precision negatively. However, the “limited” option of Step (2.b.) had a better precision‐recall trade‐off than the “near‐exhaustive” option (2.a.) and therefore was used in the procedure as the component for subsequent testing, improving the FM to 90.63%.

In Step 3, the use of Germanic and Jewish names (3.a.) led to a significant increase in false‐positive results, and this component was not used in the procedure. Changes in FM after identifying Baltic and Romanian surnames (3.c.) were negligible, which might indicate the very low popularity of these types of names, and this component was not used either. The lists of substantival Russian names (3.b.) and irregular names associated with top scientists (3.d.) did not significantly affect precision, but positively affected recall and were therefore used in the subsequent testing. Overall, the combined results of the third step of the procedure improved the FM to 93.16%.

In the final step, the use of a list of Russian given names to exclude false‐positive results led to an increase of precision at the expense of recall but also improved the FM. The maximized FM of all four steps and five components of the surname‐based identification procedure amounted to 96.2%. The procedure correctly identified 940 of the 1,000 Russian names in the control dataset and captured 18 false‐positive results.

### *Application of the Procedure to the Complete ORCID Dataset*

The surname‐based identification procedure returned high‐validity results when applied in a controlled dataset. We applied the best‐performing variant of the procedure in the entire dataset of ORCID users to distinguish Russian names and, by proxy, ORCID users with Russian heritage. The purpose was twofold. First, we sought to indirectly estimate how well the procedure could identify a minority class in an imbalanced dataset. Second, we aimed to demonstrate the use and usefulness of surname‐based identification by distinguishing five subsets of Russian surname holders and outlining research problems in bibliometrics that might be addressed. One way to infer heritage beyond affiliation address is to use a tool to explore the research patterns of scientists, their mobility patterns, and collaboration network patterns. To illustrate some of these opportunities, we distinguished the following categories of ORCID users:

1. ORCID users with addresses in Russia who may have reported an international address in their career history;
2. ORCID users who reported Russian addresses, but no international addresses;
3. ORCID users who reported an international address and may have reported a Russian address;
4. ORCID users who reported at least one international and one Russian address;
5. ORCID users who reported an international address, but no Russian address.

For each of the five categories, we identified the number of scientists whose names suggest Russian heritage (see Table 3).

**Table 3:** Number of Russian heritage researchers for different types of ORCID users.

Category of ORCID user	Total users in the group	Russian heritage users
(1) ORCID users with addresses in Russia who may have reported an international address in their career history	8,799	7,501 (85.25%)
(2) ORCID users who reported Russian addresses, but no international addresses	7,378	6,559 (88.90%)
(3) ORCID users who reported an international address and may have reported a Russian address	287,484	8,561 (2.98%)
(4) ORCID users who reported at least one international and one Russian address	1,473	994 (67.48%)
(5) ORCID users who reported an international address, but no Russian address	286,030	7,718 (2.7%)

*Note*. Numbers are calculated as a percentage of total users in each group (source: ORCID, calculations by the authors. *N* = 294,746).

Insights from categories 1, 2, and 4 provide an idea of the extent of the internationalization of Russian science. Of ORCID users with mobility experience to or from Russia (Category 4), 70% have Russian names. This implies that most of these mobile scientists are Russian researchers who have traveled abroad. Among the scientists in Russia, almost 90% of those who have never been mobile have Russian names, implying Russian heritage. This finding supports our earlier assumption about the very limited numbers of foreign‐born students and researchers in the Russian research system. By combining insights from Categories 1 and 4, we can estimate that the number of overseas scientists who come to Russia to work or study does not exceed 5.5% of the total number of scientists in the country. The Russian research system appears to be highly nationally bounded and does not have a high presence of international researchers.

Categories 3, 4, and 5 provide insights into researchers with Russian heritage working outside Russia. Comparing the number of Russian heritage ORCID users identified in Category 3 with the number in Category 2, we estimate that the number of researchers with Russian heritage employed abroad equals to, and even exceeds, the number of researchers working only in Russia, at least in terms of representation in the ORCID database. When applied in publication or patent datasets, this comparison can be used to indicate the extent of brain drain from Russia.

Category 4 identifies Russian heritage scientists who are considered “first‐generation” diaspora: they received training and worked both in Russia and abroad and contribute to the knowledge exchange both in their home and host countries. Category 5 identifies “second‐generation” diaspora: ORCID users with Russian names who never reported affiliations in Russia. However, they may have retained informal networks and ties with Russia, which could be enabled for international knowledge brokerage.

## Discussion and Conclusions

The application of the surname‐based identification methods has to date been limited because of inaccurate dictionary‐based surname classification techniques and the lack of validity testing opportunities. Using lexicological morphology of surnames to infer the heritage of surname holders demonstrated superior performance, in contrast to most dictionary‐based methods, in identifying Russian heritage researchers. As illustrated in Table 3, the simplest rule‐based identification provided an *F*‐measure of more than 85%, increasing to more than 96% in a four‐step sequence. To date, Russian surnames have rarely been explored in onomastics research. This article serves as a reference material for further uses of this method in other datasets. We also offer conceptual contributions by distinguishing ethnicity and heritage and by outlining a research agenda for the further use of this method.

ORCID as a source of metadata will become more relevant in bibliometric research as its adoption becomes more widespread. For now, the use of name‐based identification helps to address the gaps in the ORCID coverage in research where ORCID is used as a repository of CV‐like data. Applying the procedure to identify the Russian heritage in the ORCID also shows institutional influences on researcher strategies. For example, our finding about the number of ORCID users with Russian heritage who work outside Russia being comparable with the number of ORCID users in Russia is likely because of the lower rates of ORCID penetration in Russia than in Europe and the USA, not because the size of Russian overseas diaspora is similar to the size of the domestic research system.

The general principles of the morphology‐based method outlined in this article can be tailored to identify a variety of types of ethnic and national heritage. When used correctly, the method demonstrates superior performance in identifying names within traditions with strong morphological regularities, such as Finnish, Greek, Japanese, Vietnamese, or Turkish (Table 4). For other ethnic and national groups, the limited or conditional use of the method can be envisioned: it may perform better in assigning names to US ethnic categories than dictionary‐based methods and could make distinctions within those categories. The method could also account for variable, but morphologically regular surnames occurring within a country, such as French‐Canadian names. The method could also be applicable to somewhat morphologically regular naming traditions, such as Indian, jointly with dictionaries and first‐name data to ensure reliable identification.

**Table 4:** Further applicability of the surname morphology method to identify heritage.

Applicable		Partially applicable
Japanese	Turkish	Indian
Finnish	German	US ethnicities and distinctions within groups
Iranian	Vietnamese	French and French Canadian
Italian	Estonian	Portuguese (in Europe only)
Greek		Nigerian (varied across ethnic groups)

Inferring heritage from name data conceptually separates heritage and affiliation history in bibliometric datasets. This methodological innovation enables exploration, on the aggregate level, of at least four areas of research, where previously the use of bibliometric tools has been limited.

First, the approach has utility for studies of scientific mobility. There are increases in both the temporary and the permanent international mobility of scientists, with implications for national research systems and for how knowledge communities interact (Archibugi & Filippetti, 2015). Conventional approaches in bibliometric studies of mobility take an author's affiliation as a proxy of their country of origin. Inferring heritage from name data instead could help to better identify the country of origin, which in turn enables distinguishing between different mobility steps in a research career of a scientist, between transborder crossing of domestic and foreign‐born scientists, and between return and onward mobility.

Second, as global scientific collaboration links are becoming denser, a growing area of research is concerned with how these networks are built, organized, and sustained. In certain settings, it has been found that shared heritage facilitates corecruiting (Tanyildiz, 2015), transnational entrepreneurship (Saxenian, 2002), and ethnic collaboration (Jin, Rousseau, Suttmeier, & Cao, 2007). Currently, most insights rely on survey data, and some hypotheses about the roles of mobile persons with hybrid heritage in transnational networks remain untested. For example, it is difficult to distinguish second‐generation scientists and inventors from first‐generation migrants. Heritage, in general cultural terms, persists through generations (Khadria, 1999). There is interest in and hypotheses about the potential ability of second‐generation scientists to play a connecting role in transnational collaborations thus facilitating skills and knowledge transfer. However, to date, these persons have been difficult to identify in bibliometric datasets.

Third, mixtures of heritage might facilitate creativity and innovativeness. Current studies provide strong support for cultural, ethnic, and gender diversity as predictors of innovation (Florida, Mellander, & Stolarick, 2008; Heinze, Shapira, Rogers, & Senker, 2009). However, aggregate‐level applications have been limited because of the lack of reliable ways to infer the heritage of scientists, inventors, and entrepreneurs. Name data could be used as an indicator of heritage, even in datasets lacking other cultural diversity markers.

Finally, heritage might influence the behavior of scientists with regard to their overall career strategies, choice of collaborators, research topics, and the frequency and outlets of outputs. These considerations underpin research on “scientific diasporas” (Séguin, Singer, & Daar, 2006), but so far only with respect to how academics communicate with their country of origin.

Concerning limitations, surname morphology‐based identification ability is influenced by both the properties of the dataset and the properties of the target population. The method developed in this article might only be effective where the surname morphology of the target population is structured. Dictionaries have advantages for less variable names. Even if regularities are present, a limitation of country and language with which the naming patterns are associated should be examined to ascertain whether heritage can be inferred reliably.

Concerning Russian surnames, a limitation is the conflation with naming traditions from Eastern European and former Soviet Central Asian countries. In bibliometric datasets, the margin of error does not seem to be significant, because Russia's population and research outputs greatly exceed those of other Slavic and post‐Soviet countries. However, this method alone cannot be used to distinguish Russian surnames from Slavic surnames.

Regarding the bias of results, first‐name data were unavailable for some ORCID user records, which may have led to the exclusion of a portion of true‐positive results. However, we used first‐name data to address regional bias specific to the Russian naming tradition to control precision, and the trade‐off was justified in the imbalanced dataset.

The main limitation of ORCID as a source of data is its self‐reported nature. Despite a growing user database, participation in ORCID varies by country, discipline, and scientific age. Russia's research outputs represent more than 6% of the global total, whereas ORCID users with addresses in Russia were less than 3%, which implies that a substantial proportion of research outputs produced in Russia are not linked to ORCID identifiers (Bohannon, 2017). ORCID profiles can also be made private. Despite its great promise, this database cannot yet be used to conduct large‐scale studies with generalizable implications for countries or organizations (see also Youtie et al., 2017).

Although this article has limitations, the use of the method presented here does open up strategies for surname‐based identification of the heritage of certain ethnic and national groups that previously have been difficult to identify with precision. Different naming traditions reveal varying amounts of information about their name holders. Deploying such surname‐based procedures as a means to distinguish the heritage of groups of scientists, along with how their heritage influences their research approaches and networks, can shed new light on how researchers work, collaborate, and move in an interconnected and globalizing world.

## Supporting information

Appendix S1: Supporting Information

## Footnotes

1 ORCID is part of the International Standard name identifier as defined by the International Standard ISO 27729:2012.

2 RSCI is a Web of Science index of more than 2000 Russian journals, developed in collaboration with the Scientific Electronic Library (elibrary.ru).

3 Rules and dictionaries developed in this work are in Supporting Information Appendix, which is available separately.

## APPENDIX 1

**Table A1:** Proportionate undersampling strategy of non‐Russian names for the control set.

Country	Share in the ORCID dataset	Author names sampled
USA	21.79%	217
Great Britain	10.41%	104
Spain	8.13%	81
Brazil	6.18%	61
India	5.85%	58
Italy	5.20%	52
Canada	4.88%	49
Australia	3.9%	39
Portugal	3.58%	36
Germany	3.25%	33
France	2.93%	29
Canada	2.28%	23
Sweden	2.28%	23
South Korea	1.95%	19
Others	14.47%	158
Total	100%	1000
