# Supplementary material for: Identifying author heritage using surname data: An application for Russian surnames
Source: J Assoc Inf Sci Technol. 2019 Jan 25;70(5):488–98. doi: 10.1002/asi.24104 (PMC6853192; doi:10.1002/asi.24104)
Supplement: Supplementary file 1 — Appendix S1: Supporting Information [file ASI-70-488-s001.docx]

**SUPPLEMENTARY MATERIAL**

**Step-by-Step Composition of steps of the surname-based identification procedure.**

For the sequence and the eventual selection of the steps, refer to Figure 1 in the publication.

**Step 1.a.** 'Base Rule' Russian Lexicological Morphology Suffixes and Endings (Source: authors, based on Unbegaun [1972])

| **Type** | **Male Versions** | **Female Versions** | **Spelling Variations** |
| --- | --- | --- | --- |
| Patronymic / metronymic - general | ov; ev; ko; cyn; in; icha; kach; nich; vich; vik; yuk | ova; eva; cyna; ina; | euv; gyn; tsin; tsyn; tsyk; ruk; chuk; |
| Patronymic - Adjectival | ii; noy; voy; ych; yi; yok; ysh | naya; vaya; | nay; ij; kih; naia; noi; vaia; voi; yj; ykch; ykh; yy; ikh; |
| Patronymic - Geographical | skii; skoi | skaya; chaya; tkaya | chaia; skaia; skaiya; skaj; skaja; skij; skiy; skoia; sky; tkaia |

**Step 1.b.** Popular surnames (source: authors, based on Wikipedia [2017])

| Abakumov | Ernet | Kulakova | Popov | Suvorova |
| --- | --- | --- | --- | --- |
| Abakumova | Erneta | Kulibin | Popova | Svalov |
| Abalyshev | Eshman | Kulibina | Popyrin | Svalova |
| Abalysheva | Eshmana | Kulik | Popyrina | Syanov |
| Abarnikov | Esmond | Kulika | Portnov | Syanova |
| Abarnikova | Esmonda | Kulikov | Portnova | Sychkin |
| Abdulov | Essen | Kulikova | Posokhov | Sychkina |
| Abdulova | Essena | Kuptsov | Posokhova | Sychyov |
| Abramov | Estse | Kuptsova | Post | Sychyova |
| Abramova | Estsea | Kurakin | Posta | Syomin |
| Abramovich | Etush | Kurakina | Potapov | Syomina |
| Abrankovich | Etusha | Kurbatov | Potapova | Sysoyev |
| Adaksin | Eybozhenko | Kurbatova | Potrepalov | Sysoyeva |
| Adaksina | Eybozhenkoa | Kurchin | Potrepalova | Sytnikov |
| Afanasyev | Fadeev | Kurchina | Potyomkin | Sytnikova |
| Afanasyeva | Fadeeva | Kurdin | Potyomkina | Syukosev |
| Afonin | Fammus | Kurdina | Pozdnyakov | Syukoseva |
| Afonina | Fammusa | Kurepin | Pozdnyakova | Tabakov |
| Agafonov | Famusov | Kurepina | Pozharskaya | Tabakova |
| Agafonova | Famusova | Kurganov | Pozharsky | Tabernakulov |
| Agapov | Fanin | Kurganova | Prazdnikov | Tabernakulova |
| Agapova | Fanina | Kuritsyn | Prazdnikova | Talalikhin |
| Ageykin | Fedchenkov | Kuritsyna | Preobrazhenskaya | Talalikhina |
| Ageykina | Fedchenkova | Kurochkin | Preobrazhensky | Talanov |
| Agliullin | Fedin | Kurochkina | Pribylov | Talanova |
| Agliullina | Fedina | Kurpatov | Pribylova | Tamakhin |
| Akhremenko | Fedoseyev | Kurpatova | Prikhodko | Tamakhina |
| Akinfeev | Fedoseyeva | Kursalin | Prikhodkoa | Tamarkin |
| Akinfeeva | Fedosov | Kursalina | Primakov | Tamarkina |
| Aksakov | Fedosova | Kurtashkin | Primakova | Tankov |
| Aksakova | Fedotov | Kurtashkina | Privalov | Tankova |
| Aksenchuk | Fedotova | Kustov | Privalova | Tarasov |
| Aksenchuka | Fedulov | Kustova | Prokhorov | Tarasova |
| Aksyonov | Fedulova | Kutepov | Prokhorova | Tarnovetskaya |
| Aksyonova | Fekhlachev | Kutepova | Pronichev | Tarnovetsky |
| Akulov | Fekhlacheva | Kutikov | Pronicheva | Tatarinov |
| Akulova | Fenenko | Kutikova | Pronin | Tatarinova |
| Alenin | Fenenkoa | Kutuzov | Pronina | Tatarintsev |
| Alenina | Fetisov | Kutuzova | Proskurkin | Tatarintseva |
| Alexandrov | Fetisova | Kutyakov | Proskurkina | Tatarov |
| Alexandrova | Filatov | Kutyakova | Protasov | Tatarova |
| Alexeyev | Filatova | Kuvayev | Protasova | Tataurov |
| Alexeyeva | Filchenkov | Kuvayeva | Pshenichnikov | Tataurova |
| Aleyev | Filchenkova | Kuzkin | Pshenichnikova | Tattar |
| Aleyeva | Filenkov | Kuzkina | Pudin | Tattara |
| Alistratov | Filenkova | Kuzmich | Pudina | Taushev |
| Alistratova | Filimonov | Kuzmicha | Pudovkin | Tausheva |
| Aliyev | Filimonova | Kuzmin | Pudovkina | Telitsyn |
| Aliyeva | Filipov | Kuzmina | Pugachyov | Telitsyna |
| Alliluyev | Filipova | Kuznetsov | Pugachyova | Tepliashin |
| Alliluyeva | Filippov | Kuznetsova | Pugin | Tepliashina |
| Alogrin | Filippova | Kuzubov | Pugina | Tepliashina |
| Alogrina | Firsov | Kuzubova | Puldov | Tepliashinaa |
| Alyokhin | Firsova | Kvasov | Puldova | Teplov |
| Alyokhina | Flyorov | Kvasova | Pushkaryov | Teplova |
| Amaliyev | Flyorova | Lachinov | Pushkaryova | Terebov |
| Amaliyeva | Fokin | Lachinova | Pushkin | Terebova |
| Amelin | Fokina | Lachkov | Pushkina | Terekhov |
| Amelina | Fomenkov | Lachkova | Pushnoy | Terekhova |
| Aminev | Fomenkova | Lagoshin | Pushnoya | Tereshchenko |
| Amineva | Fomichyov | Lagoshina | Putilin | Tereshchenkoa |
| Ananyev | Fomichyova | Lagranskaya | Putilina | Teryoshin |
| Ananyeva | Fomin | Lagransky | Putilov | Teryoshina |
| Anasenko | Fomina | Lagutov | Putilova | Teterev |
| Anasenkoa | Fonvizin | Lagutova | Putin | Tetereva |
| Andreyev | Fonvizina | Lantsov | Putina | Tikhokhod |
| Andreyeva | Foroponov | Lantsova | Putinov | Tikhokhoda |
| Andreyushkin | Foroponova | Lapayev | Putinova | Tikhomirov |
| Andreyushkina | Franko | Lapayeva | Putyatin | Tikhomirova |
| Andronikov | Frankoa | Lapidus | Putyatina | Tikhonenko |
| Andronikova | Frantsev | Lapidusa | Puzakov | Tikhonenkoa |
| Andropov | Frantseva | Lapin | Puzakova | Tikhonov |
| Andropova | Frolov | Lapina | Puzanov | Tikhonova |
| Andryukhin | Frolova | Lapotnikov | Puzanova | Tikhvinskaya |
| Andryukhina | Fukin | Lapotnikova | Pyanykh | Tikhvinsky |
| Anikanov | Fukina | Laptev | Pyanykh | Timofeyev |
| Anikanova | Furmanov | Lapteva | Pyatosin | Timofeyeva |
| Anikin | Furmanova | Lapukhov | Pyatosina | Timoshenko |
| Anikina | Fyodorov | Lapukhova | Pyryev | Timoshenkoa |
| Anishin | Fyodorova | Lapunov | Pyryeva | Timoshkin |
| Anishina | Fyokhlachev | Lapunova | Pyzhalov | Timoshkina |
| Anisimov | Fyokhlacheva | Larin | Pyzhalova | Tipalov |
| Anisimova | Gachev | Larina | Rabinovich | Tipalova |
| Ankudinov | Gacheva | Larionov | Rabinovicha | Titov |
| Ankudinova | Gagarin | Larionova | Rabrenovich | Titova |
| Annikov | Gagarina | Laskutin | Rabrenovicha | Tkachenko |
| Annikova | Gagolin | Laskutina | Rafikov | Tkachenkoa |
| Anokhin | Gagolina | Lavrentyev | Rafikova | Tkachyov |
| Anokhina | Galdin | Lavrentyeva | Ramazanov | Tkachyova |
| Anoshkin | Galdina | Lavrov | Ramazanova | Tokarev |
| Anoshkina | Galerkin | Lavrova | Raskalov | Tokareva |
| Anosov | Galerkina | Lazarev | Raskalova | Tokmakov |
| Anosova | Galiaskarov | Lazareva | Raspopov | Tokmakova |
| Anrep | Galiaskarova | Lebedev | Raspopova | Tolbanov |
| Anrepa | Galkin | Lebedeva | Rasputin | Tolbanova |
| Antakov | Galkina | Lebedinskaya | Rasputina | Tolkachyov |
| Antakova | Galygin | Lebedinsky | Rasskazov | Tolkachyova |
| Antipin | Galygina | Lebedintsev | Rasskazova | Tolmachyov |
| Antipina | Garanin | Lebedintseva | Rastorguyev | Tolmachyova |
| Antipov | Garanina | Ledovskoy | Rastorguyeva | Tolokonskaya |
| Antipova | Garifullin | Ledovskoya | Ratkevich | Tolokonsky |
| Antonov | Garifullina | Legkodimov | Ratkevicha | Tolstobrov |
| Antonova | Garin | Legkodimova | Rayt | Tolstobrova |
| Antonovich | Garina | Lel | Rayta | Tolstokozhev |
| Antonovicha | Gavrikov | Lela | Razin | Tolstokozheva |
| Apalkov | Gavrikova | Lelukh | Razina | Tolstoy |
| Apalkova | Gavrilenkov | Lelukha | Razuvayev | Tolstoya |
| Aptekar | Gavrilenkova | Leonidov | Razuvayeva | Toporkov |
| Aptekara | Gavrilov | Leonidova | Rementin | Toporkova |
| Ardankin | Gavrilova | Leonov | Rementina | Toporov |
| Ardankina | Genkin | Leonova | Remizov | Toporova |
| Arefyev | Genkina | Lepyokhin | Remizova | Torchinovich |
| Arefyeva | Gerasimov | Lepyokhina | Repin | Torchinovicha |
| Aristarkhov | Gerasimova | Lermontov | Repina | Toropov |
| Aristarkhova | Gibazov | Lermontova | Reshetilov | Toropova |
| Aristov | Gibazova | Leshchyov | Reshetilova | Traktirnikov |
| Aristova | Gilyov | Leshchyova | Reshetnikov | Traktirnikova |
| Arkhangelskaia | Gilyova | Leshev | Reshetnikova | Trapeznikov |
| Arkhangelskaya | Glagolev | Lesheva | Retyunskikh | Trapeznikova |
| Arkhangelsky | Glagoleva | Leskov | Retyunskikha | Travkin |
| Arsenyev | Glazkov | Leskova | Revyagin | Travkina |
| Arsenyeva | Glazkova | Lesnichy | Revyagina | Travnikov |
| Arshavin | Glebov | Lesnichya | Revyakin | Travnikova |
| Arshavina | Glebova | Letov | Revyakina | Tredyakovskaya |
| Artamonov | Glinin | Letova | Rezansov | Tredyakovsky |
| Artamonova | Glinina | Levin | Rezansova | Tretyakov |
| Artemyev | Globa | Levina | Reznikov | Tretyakova |
| Artemyeva | Globaa | Levkin | Reznikova | Trifonov |
| Artyomov | Glukhov | Levkina | Roborovskaya | Trifonova |
| Artyomova | Glukhova | Lidin | Roborovsky | Trofimov |
| Arzamastsev | Golodyayev | Lidina | Rodchenko | Trofimova |
| Arzamastseva | Golodyayeva | Likhachyov | Rodchenkoa | Trufanov |
| Aslakhanov | Golov | Likhachyova | Rodin | Trufanova |
| Aslakhanova | Golova | Lilov | Rodina | Trukhin |
| Aslanov | Golovakha | Lilova | Rodzyanko | Trukhina |
| Aslanova | Golovakhaa | Limonov | Rodzyankoa | Trusov |
| Aspidov | Golovanov | Limonova | Rogachyov | Trusova |
| Aspidova | Golovanova | Lipin | Rogachyova | Trutnev |
| Assonov | Golovin | Lipina | Rogov | Trutneva |
| Assonova | Golovina | Lipov | Rogova | Tryndin |
| Astafyev | Golovkin | Lipova | Rogozin | Tryndina |
| Astafyeva | Golovkina | Lisitsyn | Rogozina | Tsaplin |
| Astakhov | Golubev | Lisitsyna | Rokossovskaya | Tsaplina |
| Astakhova | Golubeva | Lisov | Rokossovsky | Tsaregorodtsev |
| Astankov | Golubkin | Lisova | Romanov | Tsaregorodtseva |
| Astankova | Golubkina | Listratov | Romanova | Tsaritsyn |
| Avandeyev | Golubov | Listratova | Roshchin | Tsaritsyna |
| Avandeyeva | Golubova | Listunov | Roshchina | Tsarsko |
| Avdeyev | Golubtsov | Listunova | Rostov | Tsarskoa |
| Avdeyeva | Golubtsova | Lobachevskaya | Rostova | Tsaryov |
| Avdonin | Golumbovskaya | Lobachevsky | Rostovtsev | Tsaryova |
| Avdonina | Golumbovsky | Lobachyov | Rostovtseva | Tsedlits |
| Averin | Goncharov | Lobachyova | Rozanov | Tsedlitsa |
| Averina | Goncharova | Loban | Rozanova | Tsekhanovetskaya |
| Averyanov | Goraya | Lobana | Rozhkov | Tsekhanovetsky |
| Averyanova | Gorayaa | Lobanov | Rozhkova | Tselikovskaya |
| Avilov | Gorbachyov | Lobanova | Rozovskaya | Tselikovsky |
| Avilova | Gorbachyova | Lobov | Rozovsky | Tselner |
| Avtukhov | Gorbunkov | Lobova | Rubashkin | Tselnera |
| Avtukhova | Gorbunkova | Loginov | Rubashkina | Tsereteli |
| Ayushiyev | Gorbunov | Loginova | Ruchkin | Tseretelia |
| Ayushiyeva | Gorbunova | Loginovskaya | Ruchkina | Tseydlerin |
| Azarov | Gorelov | Loginovsky | Rudavin | Tseydlerina |
| Azarova | Gorelova | Loktev | Rudavina | Tseydlits |
| Azhikelyamov | Goremykin | Lokteva | Rudin | Tseydlitsa |
| Azhikelyamova | Goremykina | Loktionov | Rudina | Tsigler |
| Azhishchenkov | Gorev | Loktionova | Rudnikov | Tsiglera |
| Azhishchenkova | Goreva | Lomonosov | Rudnikova | Tsimmerman |
| Babanin | Gorokhin | Lomonosova | Rudov | Tsimmermana |
| Babanina | Gorokhina | Lomovtsev | Rudova | Tsiolkovskaya |
| Babatyev | Gorokhov | Lomovtseva | Rugov | Tsiolkovsky |
| Babatyeva | Gorokhova | Lomtev | Rugova | Tsiryulnikov |
| Babichev | Gorostayev | Lomteva | Rumyantsev | Tsiryulnikova |
| Babicheva | Gorostayeva | Lopatin | Rumyantseva | Tsitnikov |
| Babikov | Gorshkov | Lopatina | Runov | Tsitnikova |
| Babikova | Gorshkova | Losev | Runova | Tsitsyanov |
| Babkin | Goryunov | Loseva | Rusakov | Tsitsyanova |
| Babkina | Goryunova | Losevskaya | Rusakova | Tsukanov |
| Baburin | Goviadinov | Losevsky | Rusanov | Tsukanova |
| Baburina | Goviadinova | Loshchilov | Rusanova | Tsulukidze |
| Babykin | Grachyov | Loshchilova | Ruskikh | Tsulukidzea |
| Babykina | Grachyova | Loskutnikov | Ruskikha | Tsvetayev |
| Bagrov | Grafov | Loskutnikova | Rusnak | Tsvetayeva |
| Bagrova | Grafova | Loskutov | Rusnaka | Tsvetkov |
| Bakrylov | Grankin | Loskutova | Russkikh | Tsvetkova |
| Bakrylova | Grankina | Lovzanskaya | Russkikha | Tsvetnov |
| Balabanov | Grebenshchikov | Lovzansky | Ryabkin | Tsvetnova |
| Balabanova | Grebenshchikova | Lubashev | Ryabkina | Tsvilenev |
| Balakhnov | Grekov | Lubasheva | Ryabkov | Tsvileneva |
| Balakhnova | Grekova | Lukashenko | Ryabkova | Tsyganov |
| Balakin | Greshnev | Lukashenkoa | Ryabov | Tsyganova |
| Balakina | Greshneva | Lukin | Ryabova | Tsyrinskaya |
| Balakirev | Gribanov | Lukina | Ryabtsev | Tsyrinsky |
| Balakireva | Gribanova | Lukov | Ryabtseva | Tsyrkunov |
| Balandin | Gribkov | Lukova | Ryakhin | Tsyrkunova |
| Balandina | Gribkova | Lukyanenko | Ryakhina | Tsyzyrev |
| Balashov | Gribov | Lukyanenkoa | Rybakov | Tsyzyreva |
| Balashova | Gribova | Lukyanov | Rybakova | Tukhachevskaya |
| Balsunov | Griboyedov | Lukyanova | Rybalkin | Tukhachevsky |
| Balsunova | Griboyedova | Luski | Rybalkina | Tumasov |
| Baltabev | Grigoryev | Luskia | Rychenkov | Tumasova |
| Baltabeva | Grigoryeva | Luzhkov | Rychenkova | Tupitsyn |
| Banin | Grinin | Luzhkova | Rykov | Tupitsyna |
| Banina | Grinina | Lvov | Rykova | Tupolev |
| Baranov | Grishin | Lvova | Ryndin | Tupoleva |
| Baranova | Grishina | Lyadov | Ryndina | Turbin |
| Baranovskaya | Gromov | Lyadova | Rytin | Turbina |
| Baranovsky | Gromova | Lyagushkin | Rytina | Turfanov |
| Baratynskaia | Grushanin | Lyagushkina | Ryurikov | Turfanova |
| Baratynskaya | Grushanina | Lyagushov | Ryurikova | Turgenev |
| Baratynsky | Gruzdev | Lyagushova | Ryzhanov | Turgeneva |
| Barbolin | Gruzdeva | Lyalyushkin | Ryzhanova | Turov |
| Barbolina | Gruzinskaya | Lyalyushkina | Ryzhikov | Turova |
| Barentsev | Gruzinsky | Lyamin | Ryzhikova | Tvardovskaya |
| Barentseva | Gryaznov | Lyamina | Ryzhkov | Tvardovsky |
| Barinov | Gryaznova | Lyapin | Ryzhkova | Tyannikov |
| Barinov | Gubanov | Lyapina | Ryzhov | Tyannikova |
| Barinova | Gubanova | Lyapunov | Ryzhova | Tychkin |
| Barinova | Gulin | Lyapunova | Rzhevskaya | Tychkina |
| Barkov | Gulina | Lyasin | Rzhevsky | Tyomkin |
| Barkova | Gunin | Lyasina | Sabantsev | Tyomkina |
| Barndyk | Gunina | Lyovkin | Sabantseva | Tyushnyakov |
| Barndyka | Gurkovskaya | Lyovkina | Sabitov | Tyushnyakova |
| Barsukov | Gurkovsky | Lytkin | Sabitova | Uashington |
| Barsukova | Guryanov | Lytkina | Sadovskaya | Uashingtona |
| Baryshev | Guryanova | Lyubimov | Sadovsky | Ubeysobakin |
| Barysheva | Guryev | Lyubimova | Sadykov | Ubeysobakina |
| Baryshnikov | Guryeva | Lyubimtsev | Sadykova | Ubysh |
| Baryshnikova | Gusarov | Lyubimtseva | Safiyulin | Ubysha |
| Baskin | Gusarova | Lyubov | Safiyulina | Udom |
| Baskina | Gusev | Lyubova | Safronov | Udoma |
| Batishchev | Guseva | Lyutenkov | Safronova | Uglichinin |
| Batishcheva | Gushchin | Lyutenkova | Sagadeyev | Uglichinina |
| Batrutdinov | Gushchina | Lyutov | Sagadeyeva | Uglitskaya |
| Batrutdinova | Gusin | Lyutova | Saitov | Uglitsky |
| Bazanov | Gusina | Lyzlov | Saitova | Uglov |
| Bazanova | Guskov | Lyzlova | Sakharov | Uglova |
| Bazarov | Guskova | Madulin | Sakharova | Ugolev |
| Bazarova | Guslyakov | Madulina | Salagin | Ugoleva |
| Bazhanov | Guslyakova | Mager | Salagina | Ugolnikov |
| Bazhanova | Ibragimov | Magera | Salko | Ugolnikova |
| Bazhenov | Ibragimova | Magomedov | Salkoa | Uitskaya |
| Bazhenova | Ignatkovich | Magomedova | Salkov | Uitsky |
| Bazin | Ignatkovicha | Makarov | Salkova | Ukhov |
| Bazina | Ignatyev | Makarova | Salnikov | Ukhova |
| Bebchuk | Ignatyeva | Makhmudov | Salnikova | Ukhtomskaya |
| Bebchuka | Igoshin | Makhmudova | Saltanov | Ukhtomsky |
| Bebnev | Igoshina | Maklakov | Saltanova | Uladimov |
| Bebneva | Igumnov | Maklakova | Samarin | Uladimova |
| Bekhterev | Igumnova | Maksimov | Samarina | Ulanov |
| Bekhtereva | Ikanov | Maksimova | Samokhin | Ulanova |
| Belevich | Ikanova | Maksimushkin | Samokhina | Ulitskaya |
| Belevicha | Ikashev | Maksimushkina | Samoylov | Ulitsky |
| Beliberdiyev | Ikasheva | Maksudov | Samoylova | Ulyanin |
| Beliberdiyeva | Ilkun | Maksudova | Samsonov | Ulyanina |
| Belikov | Ilkuna | Malakhov | Samsonova | Ulyanov |
| Belikova | Ilyasov | Malakhova | Sannikov | Ulyanova |
| Belinskaya | Ilyasova | Malchikov | Sannikova | Ulyashin |
| Belinsky | Ilyin | Malchikova | Sapalyov | Ulyashina |
| Belitrov | Ilyina | Malikov | Sapalyova | Umametev |
| Belitrova | Ilyukhin | Malikova | Sapogov | Umameteva |
| Belochkin | Ilyukhina | Malinin | Sapogova | Umanov |
| Belochkina | Ilyushin | Malinina | Sapozhnikov | Umanova |
| Beloglazov | Ilyushin | Malinov | Sapozhnikova | Umskaya |
| Beloglazova | Ilyushina | Malinova | Saprykin | Umsky |
| Belomestin | Ilyushina | Malykhin | Saprykina | Ungern |
| Belomestina | Ilyushkin | Malykhina | Sarnychev | Ungerna |
| Belomestnaya | Ilyushkina | Malyshev | Sarnycheva | Unkovskaya |
| Belomestnov | Inozemtsev | Malysheva | Savasin | Unkovsky |
| Belomestnova | Inozemtseva | Malyugin | Savasina | Untilov |
| Belomestny | Inshov | Malyugina | Savenkov | Untilova |
| Belomestnykh | Inshova | Mamin | Savenkova | Urakov |
| Belomestnykh | Ipatyev | Mamina | Savinkov | Urakova |
| Belomestov | Ipatyeva | Mamonov | Savinkova | Uralets |
| Belomestova | Isayev | Mamonova | Savrasov | Uraletsa |
| Belorusov | Isayeva | Mamykin | Savrasova | Urbanovskaya |
| Belorusova | Ishutin | Mamykina | Savvatimov | Urbanovsky |
| Belousov | Ishutina | Manin | Savvatimova | Urusov |
| Belousova | Ismaylov | Manina | Savvin | Urusova |
| Belov | Ismaylova | Mantorov | Savvina | Usachyov |
| Belova | Istomin | Mantorova | Say | Usachyova |
| Belyakov | Istomina | Manyakin | Saya | Usatov |
| Belyakova | Ivakin | Manyakina | Sayan | Usatova |
| Bendlin | Ivakina | Marin | Sayana | Usenko |
| Bendlina | Ivankov | Marina | Sayankin | Usenkoa |
| Benediktov | Ivankova | Marinin | Sayankina | Ushakov |
| Benediktova | Ivanov | Marinina | Sayankov | Ushakova |
| Berezhnoy | Ivanova | Marinkin | Sayankova | Usilov |
| Berezhnoya | Ivashin | Marinkina | Sayanov | Usilova |
| Berezin | Ivashina | Marinov | Sayanova | Usov |
| Berezina | Ivashov | Marinova | Sayanovich | Usova |
| Beriya | Ivashova | Markin | Sayanovicha | Usoyev |
| Beriyaa | Ivazov | Markina | Sayanskaya | Usoyeva |
| Bershov | Ivazova | Markov | Sayansky | Uspenskaya |
| Bershova | Ivchenko | Markova | Saytakhmetov | Uspensky |
| Besfamilnaya | Ivchenkoa | Martyushev | Saytakhmetova | Ustimovich |
| Besfamilny | Ivkin | Martyusheva | Sazonov | Ustimovicha |
| Beskryostnov | Ivkina | Maryin | Sazonova | Ustinov |
| Beskryostnova | Ivolgin | Maryina | Schastlivtsev | Ustinova |
| Bespalov | Ivolgina | Masharin | Schastlivtseva | Ustyuzhanin |
| Bespalova | Izhutin | Masharina | Sechenov | Ustyuzhanina |
| Bessonov | Izhutina | Mashir | Sechenova | Utterklo |
| Bessonova | Izmaylov | Mashira | Sedelnikov | Utterkloa |
| Bezborodov | Izmaylova | Maslak | Sedelnikova | Utyosov |
| Bezborodova | Izyumov | Maslaka | Sedov | Utyosova |
| Bezrodnaya | Izyumova | Maslov | Sedova | Utyuzhin |
| Bezrodny | Kabinov | Maslova | Seleznyov | Utyuzhina |
| Bezrukov | Kabinova | Masmekh | Seleznyova | Uvarov |
| Bezrukova | Kablukov | Masmekha | Selidov | Uvarova |
| Bezukladnikov | Kablukova | Masmekhov | Selidova | Vagin |
| Bezukladnikova | Kachusov | Masmekhova | Selivanov | Vagina |
| Bingerin | Kachusova | Matveyev | Selivanova | Vakhrov |
| Bingerina | Kadnikov | Matveyeva | Semerikov | Vakhrova |
| Biryukov | Kadnikova | Maysak | Semerikova | Vakhrushev |
| Biryukova | Kadtsyn | Maysaka | Semichayevskaya | Vakhrusheva |
| Blanter | Kadtsyna | Mazhulin | Semichayevsky | Valevach |
| Blantera | Kadurin | Mazhulina | Semyanin | Valevacha |
| Blatov | Kadurina | Mednikov | Semyanina | Vanzin |
| Blatova | Kalagin | Mednikova | Semyonov | Vanzina |
| Blazhenov | Kalagina | Medvedev | Semyonova | Varennikov |
| Blazhenova | Kalashnik | Medvedeva | Senkin | Varennikova |
| Blinov | Kalashnika | Medvedkov | Senkina | Varushkin |
| Blinova | Kalganov | Medvedkova | Senotrusov | Varushkina |
| Blok | Kalganova | Mekhantyev | Senotrusova | Vasilevskaya |
| Bloka | Kalinin | Mekhantyeva | Serebrov | Vasilevsky |
| Blokhin | Kalinina | Meledin | Serebrova | Vasilyev |
| Blokhina | Kalugin | Meledina | Serebryakov | Vasilyeva |
| Blokov | Kalugina | Melekhov | Serebryakova | Vasilyevykh |
| Blokova | Kalyagin | Melekhova | Sergeyev | Vasilyevykh |
| Blum | Kalyagina | Melikov | Sergeyeva | Vasin |
| Bluma | Kamenskikh | Melikova | Serov | Vasina |
| Bobkov | Kamenskikha | Melnikov | Serova | Vasnetsov |
| Bobkova | Kamkin | Melnikova | Serpionov | Vasnetsova |
| Bobr | Kamkina | Menshchikov | Serpionova | Vasnev |
| Bobra | Kanadin | Menshchikova | Seryogin | Vasneva |
| Bobrik | Kanadina | Menshikov | Seryogina | Vavilov |
| Bobrika | Kanadov | Menshikova | Severinov | Vavilova |
| Bobrinskaya | Kanadova | Merkulov | Severinova | Vazov |
| Bobrinsky | Kanadtsev | Merkulova | Severov | Vazova |
| Bobrov | Kanadtseva | Merkushev | Severova | Vedeneyev |
| Bobrova | Kanalin | Merkusheva | Sevostyanov | Vedeneyeva |
| Bocharov | Kanalina | Meshcheryakov | Sevostyanova | Vedenin |
| Bocharova | Kandinskaya | Meshcheryakova | Shabalin | Vedenina |
| Bodrov | Kandinsky | Mesyats | Shabalina | Vedernikov |
| Bodrova | Kapitsa | Mesyatsa | Shabunin | Vedernikova |
| Bogachyov | Kapitsaa | Migunov | Shabunina | Venediktov |
| Bogachyova | Kapralov | Migunova | Shagidzyanov | Venediktova |
| Bogatyryov | Kapralova | Mikhalev | Shagidzyanova | Verenich |
| Bogatyryova | Kapriyanov | Mikhaleva | Shakmakov | Verenicha |
| Bogdanov | Kapriyanova | Mikhalitsin | Shakmakova | Vereshchagin |
| Bogdanova | Kapustin | Mikhalitsina | Shalomentsev | Vereshchagina |
| Bogolepov | Kapustina | Mikhalitsyn | Shalomentseva | Vershinin |
| Bogolepova | Kapustov | Mikhalitsyna | Shalyapin | Vershinina |
| Bogolyubov | Kapustova | Mikhaylov | Shalyapina | Veselov |
| Bogolyubova | Kapylyushnaya | Mikhaylova | Shaposhnikov | Veselova |
| Bogolyubskaya | Kapylyushny | Mikheyev | Shaposhnikova | Veselovskaya |
| Bogolyubsky | Karandashov | Mikheyeva | Shapovalov | Veselovsky |
| Bogomazov | Karandashova | Milekhin | Shapovalova | Vetochkin |
| Bogomazova | Karantirov | Milekhina | Sharapov | Vetochkina |
| Bogomolov | Karantirova | Miloradov | Sharapova | Vetrov |
| Bogomolova | Karaulin | Miloradova | Sharonov | Vetrova |
| Bogrov | Karaulina | Miloslavskaya | Sharonova | Vikashev |
| Bogrova | Karaulov | Miloslavsky | Sharov | Vikasheva |
| Bogun | Karaulova | Milyukov | Sharova | Vikhrov |
| Boguna | Karavayev | Milyukova | Sharshin | Vikhrova |
| Bok | Karavayeva | Milyutin | Sharshina | Vinogradov |
| Boka | Karbainov | Milyutina | Shashlov | Vinogradova |
| Bokaryov | Karbainova | Minayev | Shashlova | Vinokurov |
| Bokaryova | Karchagin | Minayeva | Shastin | Vinokurova |
| Boldayev | Karchagina | Mineyev | Shastina | Vitayev |
| Boldayeva | Karetnikov | Mineyeva | Shatalov | Vitayeva |
| Boldyrev | Karetnikova | Minin | Shatalova | Vitsin |
| Boldyreva | Karev | Minina | Shchavelskaya | Vitsina |
| Bolotnikov | Kareva | Minkin | Shchavelsky | Vitvinin |
| Bolotnikova | Kargin | Minkina | Shchavlev | Vitvinina |
| Bolshakov | Kargina | Minkovski | Shchavleva | Vlacic |
| Bolshakova | Kariyev | Minkovskia | Shchedrin | Vlacica |
| Bolshov | Kariyeva | Mirnov | Shchedrina | Vodoleyev |
| Bolshova | Karnaukhov | Mirnova | Shchedrov | Vodoleyeva |
| Boltonogov | Karnaukhova | Mirokhin | Shchedrova | Vodovatov |
| Boltonogova | Kartashov | Mirokhina | Shchegelskaya | Vodovatova |
| Bondarchuk | Kartashova | Mironov | Shchegelsky | Vodovos |
| Bondarchuka | Kartashyov | Mironova | Shcheglov | Vodovosa |
| Bondarev | Kartashyova | Mirov | Shcheglova | Vodyanov |
| Bondareva | Karyavin | Mirova | Shchegolev | Vodyanova |
| Boreyev | Karyavina | Mirskaya | Shchegoleva | Volikov |
| Boreyeva | Karzhov | Mirsky | Shchegolikhin | Volikova |
| Borisyuk | Karzhova | Misalov | Shchegolikhina | Volkov |
| Borisyuka | Kasaty | Misalova | Shchegolyayev | Volkova |
| Borodin | Kasatya | Mishin | Shchegolyayeva | Volodin |
| Borodina | Kasharin | Mishina | Shchekochikhin | Volodina |
| Borovkov | Kasharina | Mishnev | Shchekochikhina | Volosenkov |
| Borovkova | Kashirin | Mishneva | Shchepkin | Volosenkova |
| Bortnik | Kashirina | Mishutin | Shchepkina | Voloshin |
| Bortnika | Kashirskaya | Mishutina | Shcherbakov | Voloshina |
| Bortsov | Kashirsky | Mitin | Shcherbakova | Volvakov |
| Bortsova | Kashkanov | Mitina | Shcherbatykh | Volvakova |
| Borzilov | Kashkanova | Mitkin | Shcherbatykh | Vorobyov |
| Borzilova | Kashnikov | Mitkina | Shcherbina | Vorobyova |
| Bovarin | Kashnikova | Mitrofanov | Shcherbinaa | Voronin |
| Bovarina | Kashuba | Mitrofanova | Shchetinin | Voronina |
| Boyarov | Kashubaa | Mizenov | Shchetinina | Voronkov |
| Boyarova | Kasyanenko | Mizenova | Shchetkin | Voronkova |
| Boydalo | Kasyanenkoa | Moiseyev | Shchetkina | Voronov |
| Boydaloa | Kasyanov | Moiseyeva | Shchitt | Voronova |
| Boykov | Kasyanova | Mokhov | Shchitta | Vorontsov |
| Boykova | Katayev | Mokhova | Shchukin | Vorontsova |
| Boytsov | Katayeva | Molchanov | Shchukina | Voskoboynikov |
| Boytsova | Katerinochkin | Molchanova | Shchurov | Voskoboynikova |
| Bragin | Katerinochkina | Mordvinov | Shchurova | Voskresenskaya |
| Bragina | Katin | Mordvinova | Shelagin | Voskresensky |
| Brantov | Katina | Morenov | Shelagina | Votyakov |
| Brantova | Kaverin | Morenova | Shelepov | Votyakova |
| Brezhnev | Kaverina | Moroshkin | Shelepova | Vyalitsyn |
| Brezhneva | Kazakov | Moroshkina | Shelomov | Vyalitsyna |
| Brusilov | Kazakova | Morozov | Shelomova | Vyrypayev |
| Brusilova | Kazankov | Morozova | Shelyapin | Vyrypayeva |
| Budanov | Kazankova | Moryakov | Shelyapina | Yablokov |
| Budanova | Kazantsev | Moryakova | Shepkin | Yablokova |
| Budayev | Kazantseva | Mosalev | Shepkina | Yablonev |
| Budayeva | Kazarezov | Mosaleva | Shepovalov | Yabloneva |
| Budnikov | Kazarezova | Mosin | Shepovalova | Yablonskaya |
| Budnikova | Kedrov | Mosina | Sheremetyev | Yablonsky |
| Budylin | Kedrova | Moskalev | Sheremetyeva | Yagovkin |
| Budylina | Khabalov | Moskaleva | Sherkov | Yagovkina |
| Bugakov | Khabalova | Moskvin | Sherkova | Yakimenko |
| Bugakova | Khabarov | Moskvina | Sherstov | Yakimenkoa |
| Bugaychuk | Khabarova | Mosyakov | Sherstova | Yakimov |
| Bugaychuka | Khabenskaya | Mosyakova | Shevelyok | Yakimova |
| Bugayev | Khabensky | Mozhayev | Shevelyoka | Yakovlev |
| Bugayeva | Khalipov | Mozhayeva | Shibalov | Yakovleva |
| Bukavitskaya | Khalipova | Mukhanov | Shibalova | Yakubov |
| Bukavitsky | Khalski | Mukhanova | Shigayev | Yakubova |
| Bukhalo | Khalskia | Mukhin | Shigayeva | Yakubovich |
| Bukhaloa | Khalturin | Mukhina | Shigin | Yakubovicha |
| Bukin | Khalturina | Mukhomorov | Shigina | Yakunin |
| Bukina | Khamidullin | Mukhomorova | Shikalov | Yakunina |
| Bukir | Khamidullina | Mukhortov | Shikalova | Yakushev |
| Bukira | Khanilov | Mukhortova | Shikhov | Yakusheva |
| Buklin | Khanilova | Mukhov | Shikhova | Yakushin |
| Buklina | Khanipov | Mukhova | Shikhranov | Yakushina |
| Bukov | Khanipova | Muratov | Shikhranova | Yamskikh |
| Bukova | Khantsev | Muratova | Shinskaya | Yamskikha |
| Bulgakov | Khantseva | Muravyov | Shinsky | Yanayev |
| Bulgakova | Kharitonov | Muravyova | Shirinov | Yanayeva |
| Bulygin | Kharitonova | Murogov | Shirinova | Yankov |
| Bulygina | Kharlamov | Murogova | Shirmanov | Yankova |
| Bunin | Kharlamova | Myagkov | Shirmanova | Yankovskaya |
| Bunina | Kharmats | Myagkova | Shirokov | Yankovsky |
| Burdukovskaya | Kharmatsa | Myasnikov | Shirokova | Yanukovich |
| Burdukovsky | Kharzin | Myasnikova | Shishkanov | Yanukovicha |
| Burkov | Kharzina | Myatlev | Shishkanova | Yarmolnik |
| Burkova | Khaslik | Myatleva | Shishkin | Yarmolnika |
| Burmakin | Khaslika | Myaukin | Shishkina | Yaromeyev |
| Burmakina | Khigir | Myaukina | Shishko | Yaromeyeva |
| Burov | Khigira | Myshelov | Shishkoa | Yaroslavskaya |
| Burova | Khlebnikov | Myshelova | Shishlov | Yaroslavsky |
| Burtsov | Khlebnikova | Myshkin | Shishlova | Yaroslavtsev |
| Burtsova | Khlebov | Myshkina | Shishov | Yaroslavtseva |
| Bury | Khlebova | Nabatov | Shishova | Yartsev |
| Burya | Khloponin | Nabatova | Shkuratov | Yartseva |
| Buryakov | Khloponina | Nardin | Shkuratova | Yartsin |
| Buryakova | Khmelnov | Nardina | Shkut | Yartsina |
| Buturovich | Khmelnova | Nasonov | Shkuta | Yasenev |
| Buturovicha | Khodyayev | Nasonova | Shlykov | Yaseneva |
| Butusov | Khodyayeva | Naumenko | Shlykova | Yashin |
| Butusova | Khokhlachev | Naumenkoa | Shmagin | Yashina |
| Butylin | Khokhlacheva | Naumov | Shmagina | Yashkin |
| Butylina | Kholod | Naumova | Shmakov | Yashkina |
| Buzinskaya | Kholoda | Nazarov | Shmakova | Yasneyev |
| Buzinsky | Kholodov | Nazarova | Shmelev | Yasneyeva |
| Bychkov | Kholodova | Nechayev | Shmeleva | Yaytsev |
| Bychkova | Khomkolov | Nechayeva | Shpak | Yaytseva |
| Bykov | Khomkolova | Nedelyayev | Shpaka | Yazov |
| Bykova | Khorkov | Nedelyayeva | Shpikalov | Yazova |
| Bylinkin | Khorkova | Nekrasov | Shpikalova | Yedemskaya |
| Bylinkina | Khovanskaya | Nekrasova | Shubin | Yedemsky |
| Chaadayev | Khovansky | Nekrestyanov | Shubina | Yefimov |
| Chaadayeva | Khramov | Nekrestyanova | Shubkin | Yefimova |
| Chadov | Khramova | Nemtsev | Shubkina | Yefremov |
| Chadova | Khrebtov | Nemtseva | Shukshin | Yefremova |
| Chaly | Khrebtova | Nemtsov | Shukshina | Yegorov |
| Chalya | Khromov | Nemtsova | Shuldeshov | Yegorova |
| Chapayev | Khromova | Nenashev | Shuldeshova | Yelagin |
| Chapayeva | Khrushchyov | Nenasheva | Shulga | Yelagina |
| Charkov | Khrushchyova | Nepein | Shulgaa | Yelchin |
| Charkova | Khudovekov | Nepeina | Shulgin | Yelchina |
| Chayka | Khudovekova | Nesterov | Shulgina | Yelchukov |
| Chaykaa | Khudyakov | Nesterova | Shulichenko | Yelchukova |
| Chaykovskaya | Khudyakova | Netrebov | Shulichenkoa | Yeleshev |
| Chaykovsky | Khurtin | Netrebova | Shults | Yelesheva |
| Chazov | Khurtina | Nevzorov | Shultsa | Yeliseyev |
| Chazova | Khvostovskaya | Nevzorova | Shulyov | Yeliseyeva |
| Chebotaryov | Khvostovsky | Nezhdanov | Shulyova | Yelizarov |
| Chebotaryova | Kilesso | Nezhdanova | Shursha | Yelizarova |
| Chebykin | Kilessoa | Nikiforov | Shurshaa | Yeltsin |
| Chebykina | Kipriyanov | Nikiforova | Shurshalin | Yeltsina |
| Chekhov | Kipriyanova | Nikishin | Shurshalina | Yeltsov |
| Chekhova | Kirdan | Nikishina | Shurupin | Yeltsova |
| Chekmaryov | Kirdana | Nikitin | Shurupina | Yemelin |
| Chekmaryova | Kireyev | Nikitina | Shurupov | Yemelina |
| Chekudayev | Kireyeva | Nikolayev | Shurupova | Yemelyanov |
| Chekudayeva | Kirigin | Nikolayeva | Shurygin | Yemelyanova |
| Chelomey | Kirigina | Nikonov | Shurygina | Yenin |
| Chelomeya | Kirilishen | Nikonova | Shushalev | Yenina |
| Chelomeyev | Kirilishena | Nikulin | Shushaleva | Yenotin |
| Chelomeyeva | Kirillov | Nikulina | Shustelyov | Yenotina |
| Chelomtsev | Kirillova | Nizamutdinov | Shustelyova | Yenotov |
| Chelomtseva | Kirillovskaya | Nizamutdinova | Shuvalov | Yenotova |
| Chelpanov | Kirillovsky | Norin | Shuvalova | Yerkhov |
| Chelpanova | Kirilov | Norina | Shuyskaya | Yerkhova |
| Chemeris | Kirilova | Nosachyov | Shuysky | Yerkulayev |
| Chemerisa | Kirsanov | Nosachyova | Shvedov | Yerkulayeva |
| Chendev | Kirsanova | Noskov | Shvedova | Yermakov |
| Chendeva | Kiryanov | Noskova | Shvernik | Yermakova |
| Chepurin | Kiryanova | Nosov | Shvernika | Yermilov |
| Chepurina | Kislukhin | Nosova | Sidorov | Yermilova |
| Cherenchikov | Kislukhina | Novichkov | Sidorova | Yermolayev |
| Cherenchikova | Klepak | Novichkova | Sigachyov | Yermolayeva |
| Cherepanov | Klepaka | Novikov | Sigachyova | Yermolov |
| Cherepanova | Klepakhov | Novikova | Sigalov | Yermolova |
| Cherkashin | Klepakhova | Novokshonov | Sigalova | Yermushin |
| Cherkashina | Klepin | Novokshonova | Sigayev | Yermushina |
| Cherkasov | Klepina | Novoseltsev | Sigayeva | Yerofeyev |
| Cherkasova | Klimov | Novoseltseva | Silayev | Yerofeyeva |
| Chernakov | Klimova | Nozdrin | Silayeva | Yerokhin |
| Chernakova | Klimtsov | Nozdrina | Silin | Yerokhina |
| Chernetskaya | Klimtsova | Nozdryov | Silina | Yeromeyev |
| Chernetsky | Klimushin | Nozdryova | Silivanov | Yeromeyeva |
| Chernikov | Klimushina | Nuriyev | Silivanova | Yershov |
| Chernikova | Klokov | Nuriyeva | Silvestrov | Yershova |
| Chernobrovin | Klokova | Obnizov | Silvestrova | Yeryomin |
| Chernobrovina | Knyazev | Obnizova | Simakin | Yeryomina |
| Chernomyrdin | Knyazeva | Obolenskaya | Simakina | Yerzov |
| Chernomyrdina | Kochenkov | Obolensky | Simonov | Yerzova |
| Chernov | Kochenkova | Oborin | Simonova | Yesaulov |
| Chernova | Kocheryozhkin | Oborina | Sitnikov | Yesaulova |
| Chernyavskaya | Kocheryozhkina | Ogievich | Sitnikova | Yeshevskaya |
| Chernyavsky | Kolesnikov | Ogievicha | Sivakov | Yeshevsky |
| Chernykh | Kolesnikova | Ogorodnikov | Sivakova | Yesikov |
| Chernykh | Kolesov | Ogorodnikova | Siyakayev | Yesikova |
| Chernyshyov | Kolesova | Ogurtsov | Siyakayeva | Yesipov |
| Chernyshyova | Kollerov | Ogurtsova | Siyalov | Yesipova |
| Chervyakov | Kollerova | Okulov | Siyalova | Yevdokimov |
| Chervyakova | Kolobkov | Okulova | Siyan | Yevdokimova |
| Chesnokov | Kolobkova | Olenev | Siyana | Yevseyev |
| Chesnokova | Kolomnikov | Oleneva | Siyanchuk | Yevseyeva |
| Chezhekov | Kolomnikova | Olkhovskaya | Siyanchuka | Yevstigneyev |
| Chezhekova | Kolontayev | Olkhovsky | Siyangulov | Yevstigneyeva |
| Chichikov | Kolontayeva | Omelnitskiy | Siyangulova | Yevtushenkov |
| Chichikova | Kolosov | Omelnitskiya | Siyanin | Yevtushenkova |
| Chichkanov | Kolosova | Onegin | Siyanina | Yezhov |
| Chichkanova | Koltsov | Onegina | Siyanitsa | Yezhova |
| Chigrakov | Koltsova | Onipchenko | Siyanitsaa | Yolkin |
| Chigrakova | Kolupayev | Onipchenkoa | Siyankin | Yolkina |
| Chilayev | Kolupayeva | Opokin | Siyankina | Yolkov |
| Chilayeva | Komarov | Opokina | Siyanko | Yolkova |
| Chirkash | Komarova | Oprinchuk | Siyankoa | Yozhikov |
| Chirkasha | Komissarov | Oprinchuka | Siyankov | Yozhikova |
| Chistyakov | Komissarova | Orlov | Siyankova | Yozhin |
| Chistyakova | Komolov | Orlova | Siyankovskaya | Yozhina |
| Chizhikov | Komolova | Osborn | Siyankovsky | Yozhov |
| Chizhikova | Komzin | Osborna | Siyanosov | Yozhova |
| Chkalov | Komzina | Osennykh | Siyanosova | Yubkin |
| Chkalova | Kondratyev | Osennykh | Siyanov | Yubkina |
| Chmykhov | Kondratyeva | Oshurkov | Siyanova | Yudachyov |
| Chmykhova | Kondurov | Oshurkova | Siyanovich | Yudachyova |
| Chubarov | Kondurova | Osin | Siyanovicha | Yudashkin |
| Chubarova | Kondyurin | Osina | Siyanskaya | Yudashkina |
| Chuchanov | Kondyurina | Osinov | Siyanskikh | Yudin |
| Chuchanova | Konev | Osinova | Siyanskikha | Yudina |
| Chuchumashev | Koneva | Osintsev | Siyansky | Yuditskaya |
| Chuchumasheva | Konnikov | Osintseva | Siyantsev | Yuditsky |
| Chudov | Konnikova | Osipenkov | Siyantseva | Yuferev |
| Chudova | Konovalov | Osipenkova | Siyasinov | Yufereva |
| Chugunov | Konovalova | Osminin | Siyasinova | Yugantsev |
| Chugunova | Konyakov | Osminina | Sizov | Yugantseva |
| Chukchov | Konyakova | Osokin | Sizova | Yugov |
| Chukchova | Konyashev | Osokina | Sizy | Yugova |
| Chukreyev | Konyasheva | Osolodkin | Sizya | Yukhantsev |
| Chukreyeva | Kopeykin | Osolodkina | Skorobogatov | Yukhantseva |
| Chumakov | Kopeykina | Ostaltsev | Skorobogatova | Yukhtrits |
| Chumakova | Kopsov | Ostaltseva | Skorokhodov | Yukhtritsa |
| Chupakhin | Kopsova | Ostapyuk | Skorokhodova | Yumashev |
| Chupakhina | Koptsev | Ostapyuka | Skryabin | Yumasheva |
| Chupalov | Koptseva | Ostroverkhov | Skryabina | Yumatov |
| Chupalova | Korablin | Ostroverkhova | Skumin | Yumatova |
| Chupov | Korablina | Ostrovskaya | Skumina | Yunevich |
| Chupova | Korablyov | Ostrovsky | Skuratov | Yunevicha |
| Chuprakov | Korablyova | Ovechkin | Skuratova | Yunge |
| Chuprakova | Korchagin | Ovechkin | Skvortsov | Yungea |
| Chuprin | Korchagina | Ovechkina | Skvortsova | Yunkin |
| Chuprina | Korenev | Ovechkina | Slavskaya | Yunkina |
| Chuprov | Koreneva | Ozerov | Slavsky | Yurakin |
| Chuprova | Korenyov | Ozerova | Slepynin | Yurakina |
| Churkin | Korenyova | Pakhomov | Slepynina | Yurasov |
| Churkina | Kornev | Pakhomova | Slobozhanin | Yurasova |
| Chuzhinov | Korneva | Palyulin | Slobozhanina | Yurenev |
| Chuzhinova | Korneyev | Palyulina | Sluchevskaya | Yureneva |
| Daniltsin | Korneyeva | Panarin | Sluchevsky | Yurin |
| Daniltsina | Kornilov | Panarina | Smagin | Yurina |
| Danshov | Kornilova | Panfilov | Smagina | Yurkov |
| Danshova | Korolyov | Panfilova | Smekhov | Yurkova |
| Datsishin | Korolyova | Panin | Smekhova | Yurlov |
| Datsishina | Korotayev | Panina | Smeshnoy | Yurlova |
| Davydkin | Korotayeva | Pankin | Smeshnoya | Yurnayev |
| Davydkina | Koroteyev | Pankina | Smetanin | Yurnayeva |
| Davydov | Koroteyeva | Pankiv | Smetanina | Yuryev |
| Davydova | Korotkin | Pankiva | Smirnitskaya | Yuryeva |
| Dedov | Korotkina | Pankov | Smirnitsky | Yushakov |
| Dedova | Korotkov | Pankova | Smirnov | Yushakova |
| Degtyarev | Korotkova | Pankratov | Smirnova | Yushkov |
| Degtyareva | Korovin | Pankratova | Smolin | Yushkova |
| Delov | Korovina | Papanov | Smolina | Yusupov |
| Delova | Kortnev | Papanova | Smolyaninov | Yusupova |
| Demenok | Kortneva | Paramonov | Smolyaninova | Yutilov |
| Demenoka | Koryavin | Paramonova | Smotrov | Yutilova |
| Dementyev | Koryavina | Parshikov | Smotrova | Yuvelev |
| Dementyev | Koryavov | Parshikova | Snatkin | Yuveleva |
| Dementyeva | Koryavova | Parshin | Snatkina | Zabolotnaya |
| Dementyeva | Korzhakov | Parshina | Snegiryov | Zabolotny |
| Demidov | Korzhakova | Pashin | Snegiryova | Zadornov |
| Demidova | Korzhev | Pashina | Snetkov | Zadornova |
| Denikin | Korzheva | Pashkov | Snetkova | Zadorozhnaya |
| Denikina | Kosaryov | Pashkova | Sobachkin | Zadorozhny |
| Denisov | Kosaryova | Paskhin | Sobachkina | Zakharchenko |
| Denisova | Koshechkin | Paskhina | Sobakov | Zakharchenkoa |
| Dernov | Koshechkina | Pasternak | Sobakova | Zakharin |
| Dernova | Koshelev | Pasternaka | Sobchak | Zakharina |
| Derzhavin | Kosheleva | Pastukh | Sobchaka | Zakharov |
| Derzhavina | Koshkin | Pastukha | Sobolev | Zakharova |
| Desyatkov | Koshkina | Patrushev | Soboleva | Zakrevskaya |
| Desyatkova | Koshkov | Patrusheva | Sobolevskaya | Zakrevsky |
| Dezhnyov | Koshkova | Paulkin | Sobolevsky | Zakryatin |
| Dezhnyova | Koskov | Paulkina | Sochinskaya | Zakryatina |
| Dmitriyev | Koskova | Pavlenko | Sochinsky | Zaporozhets |
| Dmitriyeva | Kosma | Pavlenkoa | Sokolov | Zaporozhetsa |
| Dobrolyubov | Kosmaa | Pavlov | Sokolova | Zarubin |
| Dobrolyubova | Kosomov | Pavlova | Soldatov | Zarubina |
| Dobronravov | Kosomova | Pechenikov | Soldatova | Zaslavskaya |
| Dobronravova | Kosorukov | Pechenikova | Solodnikov | Zaslavsky |
| Dobrynin | Kosorukova | Pechkin | Solodnikova | Zavragin |
| Dobrynina | Kosterlovovich | Pechkina | Solodskikh | Zavragina |
| Dolgorukov | Kosterlovovicha | Pelevin | Solodskikha | Zavrazhin |
| Dolgorukova | Kostin | Pelevina | Solomakhin | Zavrazhina |
| Dolzhikov | Kostina | Pelyovin | Solomakhina | Zavrazhnaya |
| Dolzhikova | Kostomarov | Pelyovina | Solomin | Zavrazhnov |
| Domashev | Kostomarova | Penkin | Solomina | Zavrazhnova |
| Domasheva | Kosyak | Penkina | Solomonov | Zavrazhny |
| Domnin | Kosyaka | Perekhvatkin | Solomonova | Zavrazin |
| Domnina | Kotov | Perekhvatkina | Solovyov | Zavrazina |
| Dorofeyev | Kotova | Perestoronin | Solovyova | Zavyalov |
| Dorofeyeva | Kovalenko | Perestoronina | Somov | Zavyalova |
| Dorokhin | Kovalenkoa | Perevalov | Somova | Zaytsev |
| Dorokhina | Kovalevskaya | Perevalova | Sonin | Zaytseva |
| Dorokhov | Kovalevsky | Pereverzev | Sonina | Zefirov |
| Dorokhova | Kovalyov | Pereverzeva | Sopov | Zefirova |
| Dostoevskaya | Kovalyova | Perevyortov | Sopova | Zhabin |
| Dostoevsky | Kovrov | Perevyortova | Sorokin | Zhabina |
| Dostovalov | Kovrova | Perezhogin | Sorokina | Zharkov |
| Dostovalova | Kovshevnikov | Perezhogina | Soverighn | Zharkova |
| Dragomirov | Kovshevnikova | Perfilyev | Soverighna | Zharykhin |
| Dragomirova | Kovshutin | Perfilyeva | Spanov | Zharykhina |
| Dragunov | Kovshutina | Perminov | Spanova | Zhdanov |
| Dragunova | Koychev | Perminova | Speranskaya | Zhdanova |
| Dresvyanin | Koycheva | Permyakov | Speransky | Zheglov |
| Dresvyanina | Kozakov | Permyakova | Spravtsev | Zheglova |
| Drotenkov | Kozakova | Perov | Spravtseva | Zhelezkin |
| Drotenkova | Kozar | Perova | Stalin | Zhelezkina |
| Druganin | Kozara | Pervak | Stalina | Zheleznov |
| Druganina | Kozhukhov | Pervaka | Starikov | Zheleznova |
| Drugov | Kozhukhova | Pestov | Starikova | Zherdev |
| Drugova | Kozhurov | Pestova | Starodubov | Zherdeva |
| Dryagin | Kozhurova | Petrenko | Starodubova | Zhernakov |
| Dryagina | Kozlov | Petrenkoa | Starodubtsev | Zhernakova |
| Dryomov | Kozlova | Petrov | Starodubtseva | Zhestokov |
| Dryomova | Kozlovskaya | Petrova | Staroverov | Zhestokova |
| Dubinin | Kozlovsky | Petrukhin | Staroverova | Zhidkov |
| Dubinina | Kozyrev | Petrukhina | Starovolkov | Zhidkova |
| Dubinkin | Kozyreva | Petukhov | Starovolkova | Zhiglov |
| Dubinkina | Krasnoperov | Petukhova | Startsev | Zhiglova |
| Dubolazov | Krasnoperova | Pevchikh | Startseva | Zhigunov |
| Dubolazova | Krasnov | Pevchikha | Statnik | Zhigunova |
| Dubov | Krasnova | Pevtsov | Statnika | Zhikin |
| Dubova | Krasotkin | Pevtsova | Steblev | Zhikina |
| Dubrovskaya | Krasotkina | Pichugin | Stebleva | Zhilin |
| Dubrovsky | Kravchuk | Pichugina | Stegnov | Zhilina |
| Dudin | Kravchuka | Pichushkin | Stegnova | Zhilov |
| Dudina | Krayev | Pichushkina | Stepankov | Zhilova |
| Dudko | Krayeva | Pimenov | Stepankova | Zhirenkov |
| Dudkoa | Krayevskaya | Pimenova | Stepanov | Zhirenkova |
| Dudnik | Krayevsky | Pirogov | Stepanova | Zhirov |
| Dudnika | Krivkov | Pirogova | Stepashin | Zhirova |
| Dultsev | Krivkova | Pirozhkov | Stepashina | Zhivenkov |
| Dultseva | Krivoukhov | Pirozhkova | Stepnov | Zhivenkova |
| Dumanovskaya | Krivoukhova | Pishchalnikov | Stepnova | Zholdin |
| Dumanovsky | Krivov | Pishchalnikova | Stezhenskaya | Zholdina |
| Dunayevskaya | Krivova | Pitosin | Stezhensky | Zhukov |
| Dunayevsky | Kropanin | Pitosina | Strekalov | Zhukova |
| Duranichev | Kropanina | Pivovarov | Strekalova | Zhuravlyov |
| Duranicheva | Kruchinkin | Pivovarova | Strelkov | Zhuravlyova |
| Durchenko | Kruchinkina | Plaksin | Strelkova | Zhurov |
| Durchenkoa | Kruglov | Plaksina | Strelnikov | Zhurova |
| Durov | Kruglova | Platonov | Strelnikova | Zhutov |
| Durova | Krupich | Platonova | Streltsov | Zhutova |
| Dvornikov | Krupicha | Plemyannikov | Streltsova | Zhvikov |
| Dvornikova | Krupin | Plemyannikova | Stroganov | Zhvikova |
| Dvoynev | Krupina | Poda | Stroganova | Zhzhyonov |
| Dvoyneva | Krupnov | Podaa | Subbotin | Zhzhyonova |
| Dyatlov | Krupnova | Podshivalov | Subbotina | Zigunov |
| Dyatlova | Krutikov | Podshivalova | Subotin | Zigunova |
| Dykhovichnaya | Krutikova | Pogodin | Subotina | Zimin |
| Dykhovichny | Krutin | Pogodina | Suchkov | Zimina |
| Dyogtin | Krutina | Pogodov | Suchkova | Zimnayakova |
| Dyogtina | Krutov | Pogodova | Sudlenkov | Zimnyakov |
| Dyomin | Krutova | Pogrebnov | Sudlenkova | Zinchenko |
| Dyomina | Krutoy | Pogrebnova | Sukachyov | Zinchenkoa |
| Dyrbov | Krutoya | Pokrovskaya | Sukachyova | Zlobin |
| Dyrbova | Krylov | Pokrovsky | Sukhanov | Zlobina |
| Dyuzhenkov | Krylova | Polachev | Sukhanova | Zolin |
| Dyuzhenkova | Krymov | Polacheva | Sukharnikov | Zolina |
| Ekel | Krymova | Polichev | Sukharnikova | Zolotov |
| Ekela | Krysov | Policheva | Sukhikh | Zolotova |
| Elefterov | Krysova | Polishchuk | Sukhikha | Zonov |
| Elefterova | Kryukov | Polishchuka | Sukhorukov | Zonova |
| Elkin | Kryukova | Polivanov | Sukhorukova | Zorin |
| Elkina | Kryutchkov | Polivanova | Sukin | Zorina |
| Elmpt | Kryutchkova | Polotentsev | Sukina | Zotov |
| Elmpta | Kublanov | Polotentseva | Sultanov | Zotova |
| Emanuil | Kublanova | Polovtsev | Sultanova | Zubarev |
| Emanuila | Kubyshkin | Polovtseva | Sungatulin | Zubareva |
| Emin | Kubyshkina | Poltanov | Sungatulina | Zubkov |
| Emina | Kudashov | Poltanova | Suprunov | Zubkova |
| Emskaya | Kudashova | Poltorak | Suprunova | Zubov |
| Emskikh | Kudrin | Poltoraka | Surikov | Zubova |
| Emskikha | Kudrina | Polunin | Surikova | Zuyev |
| Emsky | Kudryashov | Polunina | Surkov | Zuyeva |
| Engalychev | Kudryashova | Polushin | Surkova | Zuykov |
| Engalycheva | Kudryavtsev | Polushina | Surnin | Zuykova |
| Engelgardt | Kudryavtseva | Polyakov | Surnina | Zverev |
| Engelgardta | Kuimov | Polyakova | Suslyakov | Zvereva |
| Engovatov | Kuimova | Pomelnikov | Suslyakova | Zvyagin |
| Engovatova | Kuklachyov | Pomelnikova | Susnin | Zvyagina |
| Entin | Kuklachyova | Pomelov | Susnina | Zykin |
| Entina | Kuklev | Pomelova | Susoyev | Zykina |
| Entskaya | Kukleva | Ponchikov | Susoyeva | Zykov |
| Entsky | Kuklin | Ponchikova | Sutulin | Zykova |
| Epinger | Kuklina | Pondyakov | Sutulina | Zyomin |
| Epingera | Kuklov | Pondyakova | Suvorin | Zyomina |
| Erdeli | Kuklova | Ponikarov | Suvorina | Zyryanov |
| Erdelia | Kulagin | Ponikarova | Suvorkin | Zyryanova |
| Eristov | Kulagina | Ponomaryov | Suvorkina | Zyuganov |
| Eristova | Kulakov | Ponomaryova | Suvorov | Zyuganova |

Step 1.c. – List of Russian Surnames composed by Zhouravlev (2005)

| Abramov | Ershova | Kornilova | Nikolskya | Simonov |
| --- | --- | --- | --- | --- |
| Abramova | Evdokimov | Korolev | Nikonov | Simonova |
| Agafonov | Evdokimova | Koroleva | Nikonova | Sinitsyn |
| Agafonova | Evseev | Korol'kov | Nikulin | Sinitsyna |
| Agapov | Evseeva | Korol'kova | Nikulina | Sitnikov |
| Agapova | Fadeev | Korotkov | Noskov | Sitnikova |
| Ageev | Fadeeva | Korotkova | Noskova | Sizov |
| Ageeva | Fedorov | Korovin | Nosov | Sizova |
| Akimov | Fedorova | Korovina | Nosova | Skvortsov |
| Akimova | Fedoseyev | Korshunov | Novikov | Skvortsova |
| Aksenov | Fedoseyeva | Korshunova | Novikova | Smirnov |
| Aksenova | Fedosov | Kosarev | Odintsov | Smirnova |
| Alekhine | Fedosova | Kosareva | Odintsova | Snegirev |
| Alekhinea | Fedotov | Koshelev | Okulov | Snegireva |
| Alekseev | Fedotova | Kosheleva | Okulova | Sobolev |
| Alekseeva | Fetisov | Kostin | Oleynikov | Soboleva |
| Aleshin | Fetisova | Kostina | Oleynikova | Sofronov |
| Aleshina | Filatov | Kotov | Orekhov | Sofronova |
| Alexandrov | Filatova | Kotova | Orekhova | Sokolov |
| Alexandrova | Filimonov | Kovalev | Orlov | Sokolova |
| Ananiev | Filimonova | Kovaleva | Orlova | Solovyov |
| Ananieva | Filippov | Kozhevnikov | Osipov | Solovyova |
| Andreev | Filippova | Kozhevnikova | Osipova | Somov |
| Andreeva | Firsov | Kozin | Ostrovsky | Somova |
| Andrianov | Firsova | Kozina | Ostrovskya | Sorokin |
| Andrianova | Fokin | Kozlov | Ovchinnikov | Sorokina |
| Anikin | Fokina | Kozlova | Ovchinnikova | Sotnikov |
| Anikina | Fomichev | Kozlovskaia | Ovsyannikov | Sotnikova |
| Anisimov | Fomicheva | Kozlovskaya | Ovsyannikova | Spiridonov |
| Anisimova | Fomin | Kozlovsky | Ozerov | Spiridonova |
| Anokhin | Fomina | Kozyrev | Ozerova | Starikov |
| Anokhina | Frolov | Kozyreva | Pakhomov | Starikova |
| Antipov | Frolova | Krasnov | Pakhomova | Starostin |
| Antipova | Galkin | Krasnova | Panfilov | Starostina |
| Antonov | Galkina | Kravtsov | Panfilova | Stepanov |
| Antonova | Gavrilov | Kravtsova | Panin | Stepanova |
| Arkhipov | Gavrilova | Kruglov | Panina | Stolyarov |
| Arkhipova | Gerasimov | Kruglova | Pankov | Stolyarova |
| Artamonov | Gerasimova | Krylov | Pankova | Subbotin |
| Artamonova | Gladkov | Krylova | Pankratov | Subbotina |
| Artemov | Gladkova | Kryuchkov | Pankratova | Sudakov |
| Artemova | Glebov | Kryuchkova | Panov | Sudakova |
| Assumption | Glebova | Kryukov | Panova | Sukhanov |
| Assumptiona | Glukhov | Kryukova | Panteleev | Sukhanova |
| Astafiev | Glukhova | Kudryashov | Panteleeva | Sukharev |
| Astafieva | Glushkov | Kudryashova | Paramonov | Sukhareva |
| Astakhov | Glushkova | Kudryavtsev | Paramonova | Sukhov |
| Astakhova | Golikov | Kudryavtseva | Parfenov | Sukhova |
| Avdeev | Golikova | Kukushkin | Parfenova | Surkov |
| Avdeeva | Golovanov | Kukushkina | Pavlov | Surkova |
| Babushkin | Golovanova | Kulagin | Pavlova | Suslov |
| Babushkina | Golovin | Kulagina | Pavlovskaia | Suslova |
| Balashov | Golovina | Kulakov | Pavlovskaya | Suvorov |
| Balashova | Golubev | Kulakova | Pavlovsky | Suvorova |
| Baranov | Golubeva | Kuleshov | Petrov | Sveshnikov |
| Baranova | Goncharov | Kuleshova | Petrova | Sveshnikova |
| Barsukov | Goncharova | Kulikov | Petrovsky | Sviridov |
| Barsukova | Gorbachev | Kulikova | Petrovskya | Sviridova |
| Basov | Gorbacheva | Kupriyanov | Petukhov | Sychev |
| Basova | Gorbunov | Kupriyanova | Petukhova | Sycheva |
| Bazhenov | Gorbunova | Kurochkin | Pimenov | Tarasov |
| Bazhenova | Gordeev | Kurochkina | Pimenova | Tarasova |
| Belikov | Gordeeva | Kuzin | Pirogov | Terekhov |
| Belikova | Gorelov | Kuzina | Pirogova | Terekhova |
| Belkin | Gorelova | Kuzmin | Platonov | Terentyev |
| Belkina | Gorlov | Kuzmina | Platonova | Terentyeva |
| Belousov | Gorlova | Kuznetsov | Plotnikov | Tikhomirov |
| Belousova | Gorokhov | Kuznetsova | Plotnikova | Tikhomirova |
| Belov | Gorokhova | Lapin | Pokrovsky | Timofeev |
| Belova | Gorshkov | Lapina | Pokrovskya | Timofeeva |
| Belyaev | Gorshkova | Lapshin | Polikarpov | Titov |
| Belyaeva | Goryachev | Lapshina | Polikarpova | Titova |
| Belyakov | Goryacheva | Laptev | Polyakov | Tkachev |
| Belyakova | Goryunov | Lapteva | Polyakova | Tkacheva |
| Berezin | Goryunova | Larin | Ponomarev | Tokarev |
| Berezina | Grachev | Larina | Ponomareva | Tokareva |
| Bespalov | Gracheva | Larionov | Popov | Tolkachev |
| Bespalova | Grekov | Larionova | Popova | Tolkacheva |
| Bessonov | Grekova | Latyshev | Postnikov | Tretyakov |
| Bessonova | Gribov | Latysheva | Postnikova | Tretyakova |
| Bezrukov | Gribova | Lavrentiev | Potapov | Trifonov |
| Bezrukova | Grigoriev | Lavrentieva | Potapova | Trifonova |
| Biryukov | Grigorieva | Lavrov | Pozdnyakov | Trofimov |
| Biryukova | Grishin | Lavrova | Pozdnyakova | Trofimova |
| Blinov | Grishina | Lazarev | Prokhorov | Troitskaia |
| Blinova | Gromov | Lazareva | Prokhorova | Troitskaya |
| Blokhin | Gromova | Lebedev | Prokofiev | Troitsky |
| Blokhina | Gubanov | Lebedeva | Prokofieva | Troshin |
| Bobrov | Gubanova | Leonov | Pugachev | Troshina |
| Bobrova | Gulyaev | Leonova | Pugacheva | Tsarev |
| Bocharov | Gulyaeva | Leontiev | Rakov | Tsareva |
| Bocharova | Gurov | Leontieva | Rakova | Tsvetkov |
| Bogdanov | Gurova | Levin | Rodin | Tsvetkova |
| Bogdanova | Gusev | Levina | Rodina | Tumanov |
| Bogomolov | Guseva | Litvinov | Rodionov | Tumanova |
| Bogomolova | Gushchin | Litvinova | Rodionova | Ulyanov |
| Boldyrev | Gushchina | Lobanov | Rogov | Ulyanova |
| Boldyreva | Homyakov | Lobanova | Rogova | Ushakov |
| Bolshakov | Homyakova | Loginov | Romanov | Ushakova |
| Bolshakova | Ignatieff | Loginova | Romanova | Usov |
| Bondarev | Ignatieffa | Lopatin | Rozanov | Usova |
| Bondareva | Ignatov | Lopatina | Rozanova | Ustinov |
| Borisov | Ignatova | Losev | Rozhkov | Ustinova |
| Borisova | Ilyin | Loseva | Rozhkova | Utkin |
| Borodin | Ilyina | Lukin | Rubtsov | Utkina |
| Borodina | Ilyinsky | Lukina | Rubtsova | Uvarov |
| Bulatov | Ilyinskya | Lukyanov | Rudakov | Uvarova |
| Bulatova | Isakov | Lukyanova | Rudakova | Vasiliev |
| Bulgakov | Isakova | Lvov | Rudnev | Vasilieva |
| Bulgakova | Isayev | Lvova | Rudneva | Vavilov |
| Burov | Isayeva | Lykov | Rumyantsev | Vavilova |
| Burova | Ivanov | Lykova | Rumyantseva | Vdovin |
| Bychkov | Ivanova | Lyubimov | Rusakov | Vdovina |
| Bychkova | Izmailov | Lyubimova | Rusakova | Vereshchagin |
| Bykov | Izmailova | Makarov | Rusanov | Vereshchagina |
| Bykova | Kalachev | Makarova | Rusanova | Veshnyakov |
| Chebotarev | Kalacheva | Makeyev | Ryabinin | Veshnyakova |
| Chebotareva | Kalashnikov | Makeyeva | Ryabinina | Vinogradov |
| Cherepanov | Kalashnikova | Malakhov | Ryabov | Vinogradova |
| Cherepanova | Kalinin | Malakhova | Ryabova | Vinokurov |
| Cherkasov | Kalinina | Malinin | Rybakov | Vinokurova |
| Cherkasova | Kalmykov | Malinina | Rybakova | Vishnevskaia |
| Chernaia | Kalmykova | Maltsev | Ryzhov | Vishnevskaya |
| Chernaya | Kalugin | Maltseva | Ryzhova | Vishnevskiy |
| Chernov | Kalugina | Malyshev | Safonov | Vladimirov |
| Chernova | Kapustin | Malysheva | Safonova | Vladimirova |
| Chernyaev | Kapustina | Markelov | Sakharov | Vlasov |
| Chernyaeva | Karasev | Markelova | Sakharova | Vlasova |
| Chernyi | Karaseva | Markin | Salnikov | Volkov |
| Chernykh | Karaulov | Markina | Salnikova | Volkova |
| Chernyshev | Karpov | Markov | Samoilov | Voloshin |
| Chernysheva | Karpova | Markova | Samoilova | Voloshina |
| Chesnokov | Kartashov | Martynov | Samsonov | Vorobyov |
| Chesnokova | Kartashova | Martynova | Samsonova | Vorobyova |
| Chistyakov | Kasatkin | Maslennikov | Savelyev | Voronin |
| Chistyakova | Kasatkina | Maslennikova | Savelyeva | Voronina |
| Chizhov | Kasyanov | Maslov | Savin | Voronkov |
| Chizhova | Kasyanova | Maslova | Savina | Voronkova |
| Chumakov | Kazantsev | Matveev | Savitskaia | Voronov |
| Chumakova | Kazantseva | Matveeva | Savitskaya | Voronova |
| Davydov | Kharitonov | Maximov | Savitsky | Vorontsov |
| Davydova | Kharitonova | Maximova | Sazonov | Vorontsova |
| Degtyarev | Khokhlov | Mayorov | Sazonova | Vysotskaia |
| Degtyareva | Khokhlova | Mayorova | Scheglov | Vysotskaya |
| Dementiev | Khromov | Medvedev | Scheglova | Vysotsky |
| Dementieva | Khromova | Medvedeva | Sedov | Yakovlev |
| Demidov | Khudyakov | Melnikov | Sedova | Yakovleva |
| Demidova | Khudyakova | Melnikova | Seleznev | Yashin |
| Demin | Kireev | Merkulov | Selezneva | Yashina |
| Demina | Kireeva | Merkulova | Selivanov | Yermolov |
| Dem'yanov | Kirillov | Meshcheryakov | Selivanova | Yermolova |
| Dem'yanova | Kirillova | Meshcheryakova | Semenov | Yerofeyev |
| Denisov | Kiselev | Meshkov | Semenova | Yerofeyeva |
| Denisova | Kiseleva | Meshkova | Semin | Yezhov |
| Dmitriev | Klimov | Mikhailov | Semina | Yezhova |
| Dmitrieva | Klimova | Mikhailova | Serebryakov | Yudin |
| Dobrynin | Klyuyev | Mikheev | Serebryakova | Yudina |
| Dobrynina | Klyuyeva | Mikheeva | Sergeev | Zaitsev |
| Dolgov | Knyazev | Minaev | Sergeeva | Zaitseva |
| Dolgova | Knyazeva | Minaeva | Serov | Zakharov |
| Dorofeev | Kochergin | Minin | Serova | Zakharova |
| Dorofeeva | Kochergina | Minina | Sevastyanov | Zavyalov |
| Dorokhov | Kochetkov | Mironov | Sevastyanova | Zavyalova |
| Dorokhova | Kochetkova | Mironova | Shaposhnikov | Zelenin |
| Drozdov | Kochetov | Mitrofanov | Shaposhnikova | Zelenina |
| Drozdova | Kochetova | Mitrofanova | Shapovalov | Zharov |
| Druzhinin | Kolesnikov | Moiseev | Shapovalova | Zharova |
| Druzhinina | Kolesnikova | Moiseeva | Sharov | Zhdanov |
| Dubinin | Kolesov | Molchanov | Sharova | Zhdanova |
| Dubinina | Kolesova | Molchanova | Shcherbakov | Zhilin |
| Dubov | Kolosov | Morgunov | Shcherbakova | Zhilina |
| Dubova | Kolosova | Morgunova | Shchukin | Zhukov |
| Dubrovin | Kolpakov | Morozov | Shchukina | Zhukova |
| Dubrovina | Kolpakova | Morozova | shepherds | Zhuravlev |
| Dyakonov | Koltsov | Moskvin | shepherdsa | Zhuravleva |
| Dyakonova | Koltsova | Moskvina | Shestakov | Zimin |
| Dyakov | Komarov | Mukhin | Shestakova | Zimina |
| Dyakova | Komarova | Mukhina | Shevelev | Zinoviev |
| Efimov | Komissarov | Muratov | Sheveleva | Zinovieva |
| Efimova | Komissarova | Muratova | Shevtsov | Zlobin |
| Efremov | Kondrashov | Muraviev | Shevtsova | Zlobina |
| Efremova | Kondrashova | Muravieva | Shilov | Zolotarev |
| Egorov | Kondratov | Naumov | Shilova | Zolotareva |
| Egorova | Kondratova | Naumova | Shirokov | Zolotov |
| Eliseev | Kondratyev | Nazarov | Shirokova | Zolotova |
| Eliseeva | Kondratyeva | Nazarova | Shiryaev | Zorin |
| Elizarov | Kononov | Nechayev | Shiryaeva | Zorina |
| Elizarova | Kononova | Nechayeva | Shishkin | Zotov |
| Emelyanov | Konovalov | Nefedov | Shishkina | Zotova |
| Emelyanova | Konovalova | Nefedova | Shmelev | Zubkov |
| Eremeev | Konstantinov | Nekrasov | Shmeleva | Zubkova |
| Eremeeva | Konstantinova | Nekrasova | Shubin | Zubov |
| Eremin | Kopylov | Nesterov | Shubina | Zubova |
| Eremina | Kopylova | Nesterova | Shulgin | Zuev |
| Ermakov | Korchagin | Nikiforov | Shulgina | Zueva |
| Ermakova | Korchagina | Nikiforova | Shuvalov | Zverev |
| Ermilov | Korneev | Nikitin | Shuvalova | Zvereva |
| Ermilova | Korneeva | Nikitina | Shvetsov | Zvyagintsev |
| Ermolaev | Kornev | Nikolaev | Shvetsova | Zvyagintseva |
| Ermolaeva | Korneva | Nikolaeva | Sidorov | Zykov |
| Ershov | Kornilov | Nikolsky | Sidorova | Zykova |

Steps 2.a. and 2.b. Suffixes of Russian Surnames that originated in Soviet countries (source: authors, based on Unbegaun [1972])

| **Origin** | **Narrow / Popular (Steps 2.a. and 2.b.)** | **Broad / All (Step 2a only)** |
| --- | --- | --- |
| Ukrainian / White Russian Origin | chak; chk; chno; enka; ets; ilo; itsa; itza; juk; lik; liuk; nik; nok; okh; yak; ylo; yna; yta; zhk | ak; akh; an; ash; az; gun; ik; iv; kun; pun; run; tun; uk; ura; yz; zun |
| Armenian Origin | yan; yants; yantz |  |
| Georgian Origin | dze; shvili | eli; ali; iani; ia; ava; uri; uli; nti; ba |
| Other Origin | Iliou; iliu |  |

Step 3.a. Russian Surname Suffixes of Germanic and Jewish origin (source: authors, based on Unbegaun [1972])

| **Origin** | **Surname Suffixes** |
| --- | --- |
| Germanic | en; er; ig; ke |
| Jewish | ach; bach; burg; ein; el; ker; kind; man; mann; son |

**Step 3.b.** Irregular Russian Surnames (source: authors, based on Unbegaun and Wikipedia [2017])

| Bagmet | Dolgoplosk | Krivoglaz | Prolepo | Tarakan |
| --- | --- | --- | --- | --- |
| Barabash | Duka | Krutonog | Ptucha | Tcheverda |
| Barash | Durnovo | Krysa | Ragoza | Tharakan |
| Barbash | Dved | Kukolik | Raksha | Toman |
| Barsuk | Dymochka | Kukolnik | Razum | Trukhan |
| Belago | Dymshits | Kurbak | Rosliak | Tscherbul |
| Belan | Elago | Kvasha | Roslyak | Tsybulya |
| Belokon | Formago | Kyzyma | Rovda | Tupitso |
| Belokrys | Gazman | Lopata | Rubets | Tychino |
| Belosljud | Gogol | Lykholay | Rubetz | Varakuta |
| Beloslyud | Golovan | Maymygo | Rybas | Varavka |
| Blagovo | Golub | Maznoi | Ryzago | Veselago |
| Blayda | Humala | Maznoy | Ryzhago | Veter |
| Bober | Ivago | Medvedb | Sabodash | Voit |
| Boloban | Jusim | Mertvago | Sapgir | Volovoi |
| Bordunos | Kaplya | Molochovec | Semago | Volovoy |
| Buran | Kastyro | Moroz | Shek | Vovk |
| Buryan | Katyba | Movchan | Shkarlet | Voyt |
| Cekan | Khanakhu | Muliava | Siidra | Vremya |
| Chashei | Khitrovo | Nedobrovo | Silhanek | Yarovoi |
| Chekan | Kokh | Nevshupa | Simago | Yarovoy |
| Chekun | Koloda | Oleinik | Sivak | Zdor |
| Chernago | kolozaridi | Olejnik | Sivocha | Zhaba |
| Chernuch | Korobochka | Osak | Sivocho | Zhabo |
| Chernukha | Korvigo | Parenago | Skorik | Zhelestb |
| Chernysh | Kotseruba | Perekrest | Skorokhod | Zhuk |
| Chistopian | Kovrigo | Plochovo | Smurago | Zhuk |
| Chistopyan | Kovrigo | Plokhovo | Solodusha | Zhuzhoma |
| Chitrovo | Kozak | Poroch | Soroka | Zima |
| Chkalo | Kozel | Porokh | Strashun | Zobkalo |
| Chromec | Kozik | Portnoi | Subbota | Zozulya |
| Chudak | Krasnozhen | Portnoy | Suchodrev | Zubok |
| Delogrammatik | Krivets | Povago | Syritso |  |
| Derzhimorda | Krivetz | Prilepa | Tabakh |  |

**Step 3.c.** Russian Surname Suffixes of Baltic and Romanian Origin (source: authors, based on Unbegaun [1972])

| **Origin** | **Surname Suffixes** |
| --- | --- |
| Baltic | nek |
| Romanian | eskul |

**Step 3.d.** Irregular Names of Top-Cited Scientists (source: authors, based on RSCI [2017])

| Adjoubei | Glazman | Kozinets | Orsik | Sobol |
| --- | --- | --- | --- | --- |
| Agol | Glezer | Krainik | Palhik | Sochava |
| Akhiezer | Gokhberg | Kramida | Pamyatnykh | Sokol |
| Alshits | Goldshtein | Krasik | Pashkus | Solganik |
| Amusia | Golik | Kravets | Pasternak | Starik |
| Apel | Golimbet | Krems | Pastur | Stash |
| Asaul | Golovakha | Krichever | Patalakha | Stochik |
| Asmus | Goltsman | Krishtalik | Perepetch | Stonik |
| Azhgirey | Golub | Krongauz | Picus | Straumal |
| Babak | Gonchar | Krot | Pikuz | Strekozov |
| Babich | Gongalo | Krutik | Pinelis | Strelets |
| Bachilo | Gopich | Krylatykh | Plakida | Strelis |
| Baglai | Gorbalenya | Kryshen | Plyushchai | Stryzhak |
| Baksht | Gorban | Kryvonos | Pobylitsa | Subetto |
| Balega | Gordiets | Kuchment | Podoroga | Sukhikh |
| Barash | Gotlib | Kukes | Pokhodnya | Surai |
| Barbarash | Gotra | Kulik | Polyak | Suris |
| Barenblatt | Gourmet | Kulish | Polyan | Svergun |
| Bartsits | Govorun | Kuni | Pomogailo | Svirida |
| Baru | Granik | Kuntsman | Popel | Svitoch |
| Batyunya | Grigorash | Kuprash | Popondopulo | Tager |
| Baublis | Grinshtein | Kureichik | Poshkus | Talalay |
| Bazilevich | Gritsan | Kurlenya | Potashnik | Talzi |
| Bednyi | Gritsyuk | Kushch | Prants | Tashkun |
| Belaga | Grosberg | Kushpil | Prints | Tatur |
| Belik | Guber | Kuzhir | Psakhie | Telia |
| Belokon | Guboglo | Kuznik | Pudovik | Tkach |
| Belous | Gura | Kuzyk | Puhtel | Tolochek |
| Bert | Gurvich | Kveder | Pulinets | Tolstoy |
| Berulava | Gutsol | Kvon | Pushkar | Toperverg |
| Bezrukikh | Guz | Kyurkchan | Pustovoyt | Topuriya |
| Bibler | Guzey | Labas | Puzyr | Tretyak |
| Bilenko | Habashesku | Lachuga | Raikher | Treyvish |
| Bimad | Harkats | Landau | Raiser | Trut |
| Birshtein | Harton | Lapchik | Raizberg | Tsipis |
| Bisikalo | Havinson | Lapidus | Rapoport | Tsoi |
| Bobyr | Hodel | Lappo | Rarog | Tsvelik |
| Bohan | Holevo | Lavrik | Rashba | Tsyb |
| Bokach | Honik | Lazebnyk | Rean | Turbiner |
| Bokeriya | Hovaylo | Leflat | Reva | Turok |
| Bondar | Irzhak | Lesniak | Revich | Ulashchik |
| Bondur | Issers | Lesovik | Reznik | ulga |
| Bosak | Itkis | Levinshtein | Ritus | Untura |
| Boshno | Ivanter | Lichinitser | Rogoza | Ursul |
| Bozhok | Izaak | Liebman | Rotshtein | Vainshtein |
| Bratus | Joffe | Lifshitz | Ruban | Valentey |
| Breus | Kabachnik | Likhoded | Rubenchik | Vanag |
| Briko | Kabuzan | Linde | Rud | Vargaftik |
| Chanturia | Kagan | Lip | Rutberg | Varnek |
| Chaplik | Kaganer | Litvak | Ruuge | Vasilets |
| Chernodub | Kaleda | Livshits | Sagaidak | Vataga |
| Chernogor | Kalei | Lobach | Schvets | Vazdik |
| Chizhik | Kalyuga | Loboda | Semikoz | Veksler |
| Chokheli | Kapitsa | Lozovik | Semke | Vendik |
| Chugai | Karafet | Lukash | Serbo | Veraksa |
| Chvalun | Karasik | Lukyanets | Shagaida | Verba |
| Dadali | Karaurza | Lutsuk | Shakura | Vergun |
| Daty | Karliner | Lysak | Shalyto | Vermel |
| Dedysh | Karshenboim | Magazanik | Shaposhnik | Vershik |
| Derkach | Kashuba | Magun | Sharyi | Veselago |
| Dokshitser | Katkalo | Makhnach | Shastiko | Vinberg |
| Dolnik | Katznelson | Makshtas | Shaytura | Vinnik |
| Dreitser | Kazhava | Mamut | Shegelman | Vinokur |
| Dridze | Keilisorok | Manelis | Sheifer | Vinslav |
| Drits | Khalatur | Manikhas | Sheigal | Virovets |
| Dubonos | Khomich | Marchenya | Sheinerman | Vishik |
| Dubovik | Khotinets | Mats | Sheka | Voevoda |
| Dyeryabo | Khrapak | Mazepa | Shekhter | Vokal |
| Dygai | Khursan | Mazets | Shekhtman | Volevodz |
| Dykhne | Kibrik | Mazur | Shenkman | Volovik |
| Dykman | Kira | Mebel | Sheregi | Volpert |
| Efros | Kirichuk | Medik | Sheremet | Vorobeichik |
| Eliashberg | Kisel | Melik | Shestopal | Voropai |
| Erdes | Kivshar | Melzer | Shevel | Vul |
| Ezhela | Kizim | Merzlyak | Shkura | Vysloukh |
| Feigelan | Klyshko | Mesyats | Shliomis | Yagola |
| Feldchtein | Klyukach | Minakir | Shlotgauer | Yakhno |
| Fleishman | Knirel | Minat | Shlyakhto | Yakimets |
| Florya | Kochenda | Momot | Shneider | Yakovets |
| Frid | Kochubei | Moroz | Shnirelman | Yanbykh |
| Fridrikhson | Kodess | Mukeriya | Shoba | Yani |
| Gaidar | Kogarko | Mukha | Shoikhet | Yanz |
| Galuzo | Koifman | Mukomel | Shostak | Yarokhno |
| Gamaley | Kokoza | Mur | Shpak | Yatsun |
| Ganzhara | Koldoba | Mychka | Shpigun | Yugai |
| Garamus | Kolodnaya | Myshkis | Shreider | Yurgens |
| Garkavi | Kolomiets | Mysik | Shtark | Zaichik |
| Garmash | Komar | Naydis | Shteinman | Zaidelman |
| Geim | Konradi | Nedogoda | Shtoulberg | Zaimidoroga |
| Geltman | Koptyug | Nedolya | Shtremelb | Zarembo |
| Geltser | Korczak | Neiman | Shults | Zaruk |
| Genkal | Korenberg | Neimark | Shvartsman | Zastenker |
| Geppe | Korotkikh | Neklessa | Shvindlerman | Zavodnik |
| Gershenzon | Korzhik | Nogach | Sibiryak | Zayats |
| Gershuni | Koshelets | Novik | Sinyak | Zekhtser |
| Gerya | Koshuba | Novosad | Siptits | Zelner |
| Geshkenbein | Kostochka | Obrant | Sirota | Zherebilo |
| Gianik | Kostyrya | Odinak | Skakun | Zhevora |
| Gikal | Kotlyar | Ogar | Skamay | Zhiburt |
| Gimpelson | Kovach | Oizerman | Skoromets | Zhylich |
| Ginter | Koval | Okotrub | Skovpen | Zilber |
| Ginzburg | Kovda | Oks | Smetnik | Zlotnik |
| Gippius | Kovsh | Okun | Smilga | Zvartau |
| Gladkochub | Kovtun | Orobets | Sobelan | Zyk |

**Step 4.a.** Russian Given Names (source: authors, based on Wikipedia [2016a, 2016b])

| Aaron | Avenir | Georgiy | Maxim | Snezhana |
| --- | --- | --- | --- | --- |
| Abagor | Avenira | Georgy | Mefodii | Sobieslaw |
| Abamon | Aventin | Gerasim | Melania | Sofia |
| Abataly | Aventina | Glafira | Michail | Sofya |
| Abdaikl | Averky | Gleb | Mihail | Sonia |
| Abel | Avessalom | Gniewomir | Mijail | Sonya |
| Abelyar | Avgar | Goran | Mikhael | Sophia |
| Abijah | Avgury | Gorana | Mikhail | Sophya |
| Abily | Avgust | Gordan | Mila | Stanimir |
| Abnody | Avgusta | Gregory | Milan | Stanislav |
| Abo | Avgustin | Grigory | Milana | Stanislava |
| Abram | Avgustina | Grischa | Milena | Stefania |
| Aburom | Aviafa | Gulshat | Milica | Stella |
| Adrian | Avian | Guzel | Milorad | Stepan |
| Aelita | Avim | Helena | Milovan | Stephan |
| Afanasy | Avimelekh | Iakov | Mira | Svetlana |
| Agafa | Avip | Iana | Miroslav | Svetoslav |
| Agafangel | Avit | Ignat | Misha | Sviatoslav |
| Agafiya | Aviv | Ignatii | Mstislav | Svjatoslav |
| Agafodor | Aviva | Igor | Murat | Svyatoslav |
| Agafokliya | Avksenty | Ilar | Nadegda | Taisiya |
| Agafon | Avksily | Ilarion | Nadejda | Tamara |
| Agafonik | Avksivy | Ildar | Nadeshda | Tamaz |
| Agafonika | Avkt | Ilia | Nadezda | Tanya |
| Agap | Avlida | Ilnur | Nadezgda | Taras |
| Agapa | Avram | Ilona | Nadezhda | Tatana |
| Agapion | Avrelian | Ilya | Nadia | Tatiana |
| Agapit | Avreliya | Inal | Nail | Tatjana |
| Agapiya | Avrely | Inessa | Nastja | Tatyana |
| Agapy | Avrey | Inga | Nastya | Teimuraz |
| Agat | Avreya | Inna | Natalia | Temur |
| Agatha | Avros | iosif | Nataliia | Tikhon |
| Agav | Avsey | Iouli | Nataliya | Timofei |
| Agavva | Avtonom | Iouri | Nataly | Timofey |
| Aglaya | Avtonoma | Iraida | Natalya | Timur |
| Albert | Avudim | Irena | Natasha | Tonya |
| Albina | Avundy | Irene | Nathalie | Uliana |
| Aleksander | Avva | Irina | nest | Ulya |
| Aleksandr | Avvakir | Irma | Neven | Ulyana |
| Aleksandra | Avvakum | Iryna | Nevena | Vadim |
| Aleksei | Azaliya | Iskander | Nicholas | Vadym |
| Aleksej | Bogdan | Iurii | Nickolai | Valentin |
| Aleksey | Boris | Ivan | Nickolay | Valentina |
| Alena | Borislav | Jaroslav | Nicola | Valentyna |
| Alessya | Boyan | Joseph | Nika | Valeri |
| Alesya | Branimir | Joulia | Nikilay | Valeria |
| Alex | Branislav | Julia | Nikita | Valerie |
| Alexander | Bulat | Julie | Nikolai | Valerii |
| Alexandr | Cergej | Julija | Nikolaj | Valerij |
| Alexandra | Christina | Juri | Nikolay | Valeriy |
| Alexei | Constantin | Jury | Nikolina | Valeriya |
| Alexej | Damir | Karine | Nina | Valery |
| Alexey | Danica | Kate | Nino | Vanya |
| Alexsander | Daniel | Katerina | Nonna | Varvara |
| Alfiya | Daniil | Katherine | Novak | Vasili |
| Alice | Danil | Katja | Odetta | Vasilii |
| Aliia | Danila | Katya | Oksana | Vasilina |
| Alina | Danilo | Kira | Okxana | Vasilisa |
| Alisa | Daria | Kirill | Oleg | Vasiliy |
| Aliya | Dario | Kliment | Olesya | Vasily |
| Alla | Dariya | Klimentiy | Olga | Vassili |
| Alsu | Darko | Konstantin | Olya | Vassily |
| Alya | Darya | Konstantine | Osip | Velimir |
| Alyona | David | Kostya | Oskar | Veniamin |
| Amvrosy | Davor | Krestina | Ossip | Vera |
| Anastasia | Denis | Kristina | Oxana | Veronika |
| Anastasiia | Diana | Ksenia | Panteley | Viacheslav |
| Anastasija | Dima | Kseniya | Pavel | Viatcheslav |
| Anastasiya | Dina | Ksenya | Pavsikakiy | Victor |
| Anastassia | Dmitri | Lada | Pawel | Victoria |
| Anastassiya | Dmitrii | Lana | Peter | Viktor |
| Anastasya | Dmitriy | Larisa | Petr | Viktoria |
| Anastasyia | Dmitry | Larissa | Petya | Viktoriya |
| Anatoli | Edouard | Lena | Piotr | Violetta |
| Anatolii | Eduard | Leon | Platon | Vitali |
| Anatoly | Edward | Leonid | Polina | Vitaliy |
| Andre | Efim | Lev | Prokofiy | Vitaly |
| Andrei | Egor | Leyla | Pyotr | Vitomir |
| Andrej | Ekaterina | Leysan | Rachel | Vjacheslav |
| Andrew | Elena | Lidia | Radmila | Vlada |
| Andrey | Elina | Lidiya | Radomir | Vladan |
| Anfisa | Elizaveta | Liliya | Radoslav | Vladimir |
| Angelika | Ella | Liubov | Radovan | Vladislav |
| Anna | Elvira | Liudmyla | Radul | Volodymyr |
| Anton | Emil | Liya | Rais | Vsevolod |
| Anya | Emilb | Ljuba | Raisa | Vyachaslav |
| Anzhela | Eugen | Ljubov | Ramil | Vyacheslav |
| Anzhelika | Eugene | Lolita | Rasim | Xenia |
| Arcady | Eugenia | Lubomir | Ratimir | Yaakov |
| Arina | Eva | Lubov | Renat | Yakov |
| Arkadi | Evgeni | Ludmila | Rimma | Yana |
| Arkadiy | Evgenia | Ludmyla | Rinat | Yaroslav |
| Arkady | Evgenii | Ludomir | Rita | Yaroslaw |
| Arseni | Evgeniy | Lukyan | Rodion | Yefim |
| Arsenii | Evgeniya | Lyaisan | Roma | Yegor |
| Arseniy | Evgeny | Lydia | Roman | Yekaterina |
| Arseny | Evgueni | Lyoubov | Rostislav | Yelena |
| Artem | Ewgeni | Lyubov | Roza | Yermolay |
| Artemy | Fedor | Lyuda | Rozaliya | Yevgeniy |
| Arthur | Filipp | Lyudmila | rta | Yevgeny |
| Artur | Firuza | Magdalena | Ruslan | Yulia |
| Artyom | Fjodor | Maksim | Rustam | Yuliya |
| Asiya | Fyodor | Marat | Rustem | Yulya |
| Aurora | Galenka | Margarita | Sambor | Yuri |
| Avda | Galina | Maria | Semen | Yury |
| Avdakt | Garik | Mariia | Semyon | Zakhar |
| Avdelay | Gennadi | Marina | Serafima | Zenaida |
| Avdey | Gennadii | Mariya | Seregey | Zinaida |
| Avdifaks | Gennadij | Mark | Serge | Zinoviy |
| Avdiky | Gennadiy | Marsel | Sergei | Zinovy |
| Avdiyes | Gennadiya | Marselb | Sergej | Zoia |
| Avdon | Gennady | Marya | Sergey | Zoja |
| Avdotya | Genrikh | Masha | Serguei | Zora |
| Avel | George | Matvei | Serguey | Zoya |
| Avelina | Georgi | Matvey | Slava |  |

**References**

RSCI. (2017). Russian Science Citation Index: Author Search. Retrieved 24 August 2017, from https://elibrary.ru/authors.asp

Unbegaun, B. O. (1972). Russian Surnames. Oxford University Press.

Wikipedia. (2016a). Category: Russian masculine given names. Retrieved 17 July 2017 from https://en.wikipedia.org/w/index.php?title=Category:Russian_masculine_given_names&oldid=548046441

Wikipedia. (2016b, July 14). Category: Russian feminine given names. Retrieved 17 July 2017 from https://en.wikipedia.org/w/index.php?title=Category:Russian_feminine_given_names&oldid=729754540

Wikipedia. (2017). List of surnames in Russia. Retrieved 17 July 2017, from https://en.wikipedia.org/wiki/List_of_surnames_in_Russia
